# HSP90AA1 Knockdown Enhances Doxorubicin Sensitivity by Promoting Immunogenic Cell Death-Related Signaling and Immune-Related Tumor Microenvironment Remodeling in Breast Cancer

**DOI:** 10.3390/cancers18182913

**Published:** 2026-09-09

**Authors:** Rui Chen, Yao Jin, Chang Xie, Yan Li, Haitao Huang, Yuling Zhu, Zhibing Ming

**Affiliations:** 1 Department of Pathology, Nantong University, Nantong 226001, China; 2331310038@stmail.ntu.edu.cn; 2 Department of General Surgery, The Fourth People’s Hospital of Ningxia Hui Autonomous Region, Ningxia 750021, China; 13895410120@139.com; 3 Department of Vascular Surgery, Affiliated Nantong Clinical College of Nantong University, Nantong First People’s Hospital, Nantong 226001, China; xiechang2001@stmail.ntu.edu.cn; 4 Department of Cardiovascular Surgery, Southeast University Affiliated Nantong First People’s Hospital, Nantong 226001, China; 2113320228@stmail.ntu.edu.cn (Y.L.); 5300696@ntu.edu.cn (H.H.); 5 Department of Cardiovascular Surgery, Nantong Clinical Medical College of Kangda College of Nanjing Medical University, Nantong 226001, China; 6 Department of Vascular Surgery, Southeast University Affiliated Nantong First People’s Hospital, Nantong 226001, China

**Keywords:** HSP90AA1, breast cancer, doxorubicin response, immunogenic cell death, apoptosis, tumor immune microenvironment, antitumor immune infiltration, CD8-positive T cells

## Abstract

**Background:** Doxorubicin (DOX) is widely used in breast cancer treatment; however, therapeutic resistance and an immunosuppressive tumor microenvironment remain major obstacles to effective therapy. Immunogenic cell death (ICD) contributes to chemotherapy-induced antitumor immunity, but the molecular regulators linking DOX response, ICD-related signaling, and immune infiltration in breast cancer remain incompletely understood. This study aimed to identify and experimentally validate *HSP90AA1* as a potential regulator of DOX response and ICD-associated immune remodeling in breast cancer. **Methods:** ICD-related genes were analyzed using public breast cancer transcriptomic datasets to construct prognostic models and evaluate their associations with survival, immune infiltration, immune checkpoint expression, and HLA-related molecules. *HSP90AA1* was selected as a candidate gene for further validation. *HSP90AA1* expression was examined in breast cancer tissues and cell lines. *HSP90AA1* knockdown was performed in breast cancer cells, followed by DOX treatment. Western blotting was used to detect apoptosis-related proteins, ICD-related markers, and immune-associated molecules, including cleaved-caspase-3, BAX, BCL-2, CALR, HMGB1, p-eIF2α/eIF2α, and PD-L1. In vivo, a 4T1 syngeneic breast cancer mouse model was established to assess the effects of *HSP90AA1* knockdown combined with DOX on tumor growth and tumor immune-related protein expression. **Results:** Bioinformatic analyses showed that ICD-related molecular patterns were associated with prognosis and immune microenvironment features in breast cancer. *HSP90AA1* was upregulated in breast cancer and associated with unfavorable survival, supporting its potential relevance as an ICD-related prognostic candidate. In vitro, *HSP90AA1* knockdown enhanced DOX-induced apoptotic responses, as indicated by increased cleaved-caspase-3 and BAX expression and decreased BCL-2 expression. *HSP90AA1* knockdown also strengthened DOX-induced ICD-related molecular changes, including increased CALR, HMGB1, and p-eIF2α/eIF2α levels, accompanied by altered PD-L1 expression. In vivo, *Hsp90aa1* knockdown combined with DOX resulted in a greater reduction in terminal tumor burden than DOX treatment alone. Tumor tissues from the combination group showed increased CALR, HMGB1, CD8A, and GZMB expression, together with altered PD-L1 expression. Tumor tissues from the combination group showed increased CALR, HMGB1, CD8A, and GZMB expression, together with altered PD-L1 expression, indicating ICD-related and immune-associated molecular changes. **Conclusions:** This study identifies *HSP90AA1* as a potential ICD-related regulator of DOX response in breast cancer. *HSP90AA1* knockdown may enhance the response to DOX by promoting apoptotic responses and ICD-related molecular changes, together with immune-related tumor microenvironment remodeling. These findings provide experimental evidence supporting *HSP90AA1* as a potential therapeutic target for improving chemotherapy response in breast cancer.

## Simple Summary

Doxorubicin is an important chemotherapy drug for breast cancer, but treatment resistance and limited antitumor immune responses can reduce its effectiveness. In this study, we investigated HSP90AA1, a stress-related molecular chaperone that may help breast cancer cells adapt to chemotherapy. Using public datasets, clinical breast cancer tissues, cell experiments, and a mouse breast cancer model, we found that HSP90AA1 was highly expressed in breast cancer and was associated with unfavorable prognosis. Reducing HSP90AA1 expression enhanced the response to doxorubicin, increased apoptosis-related changes, and was accompanied by molecular changes associated with immunogenic cell death and the tumor immune microenvironment. These findings suggest that HSP90AA1 may represent a potential target for improving the response to doxorubicin in breast cancer. However, further studies are needed to determine whether this approach is effective across different breast cancer subtypes and whether it can be safely translated into clinical treatment.

## 1. Introduction

Breast cancer remains the most commonly diagnosed malignancy among women worldwide and represents a major threat to female health [1]. Despite substantial advances in surgery, endocrine therapy, targeted therapy, immunotherapy, and chemotherapy, therapeutic resistance and tumor recurrence remain important clinical challenges. Doxorubicin (DOX), an anthracycline chemotherapeutic agent, remains an important component of breast cancer treatment [2]. However, the therapeutic benefit of anthracycline-based chemotherapy varies substantially among patients, and intrinsic or acquired DOX resistance limits its long-term efficacy [3]. Therefore, identifying molecular regulators that determine DOX sensitivity may help improve chemotherapy responses and provide potential targets for combination therapy [2].

In addition to directly inducing tumor cell death, chemotherapy can also modulate antitumor immunity [4,5]. Immunogenic cell death (ICD) is a regulated form of cell death that promotes immune recognition of dying tumor cells. During ICD, tumor cells expose or release damage-associated molecular patterns, including calreticulin (CALR), high-mobility group box 1 (HMGB1), and extracellular ATP, which facilitate dendritic cell maturation and activation, antigen presentation, and cytotoxic T-cell-mediated antitumor responses [6,7]. DOX is considered a classical chemotherapeutic inducer of ICD and has been shown to promote CALR exposure, HMGB1 and ATP release, dendritic cell maturation, and CD8^+^ T-cell activation or infiltration in experimental breast cancer models [6,8]. Nevertheless, the molecular mechanisms regulating DOX-induced ICD-related signaling and the associated remodeling of the breast tumor immune microenvironment remain incompletely understood.

The tumor immune microenvironment plays a crucial role in determining treatment response and disease progression [9]. Increased infiltration of cytotoxic CD8-positive T cells and elevated expression of effector molecules such as granzyme B (GZMB) generally indicate enhanced antitumor cytolytic activity and have been associated with favorable clinical outcomes, particularly in triple-negative breast cancer [10]. Conversely, immune checkpoint signaling and immunosuppressive cell populations may facilitate tumor immune escape. For example, tumor-derived microparticles carrying programmed death-ligand 1 (PD-L1) can suppress CD8-positive T-cell activation and function, whereas FOXP3-positive regulatory T cells can inhibit antitumor immunity in triple-negative breast cancer [11,12]. Thus, PD-L1 expression and FOXP3-positive regulatory T-cell infiltration represent important immunoregulatory features of the tumor microenvironment, although their clinical implications may vary according to tumor subtype, cellular localization, spatial distribution, and treatment context [13]. Therefore, investigating the relationships among chemotherapy response, ICD-related signaling, and immune infiltration may provide new insights into breast cancer treatment [14].

Heat shock protein 90 alpha family class A member 1 (*HSP90AA1*) encodes HSP90α, a stress-inducible molecular chaperone that contributes to protein folding and stability and maintains the activity of multiple proteins involved in oncogenic signaling and cellular stress adaptation [15,16]. Aberrant *HSP90AA1* expression has been associated with breast cancer development, distant metastasis, tumor cell proliferation, migration, invasion, and unfavorable clinical characteristics [16,17]. Functionally, HSP90AA1 can support tumor cell survival and therapeutic resistance by stabilizing signaling proteins and sustaining prosurvival pathways, including AKT-related signaling [15,16]. Consistent with this role, pharmacological inhibition of HSP90 enhanced DOX-induced apoptosis in MCF-7 breast cancer cells, whereas *HSP90AA1* depletion restored DOX sensitivity in resistant breast cancer cells [18]. HSP90AA1 may also influence tumor–immune interactions, as its inhibition or silencing has been shown to reduce tumor-intrinsic resistance to cytotoxic T cells and enhance tumor-reactive CD8-positive T-cell infiltration and responses to immune checkpoint blockade in preclinical tumor models [15]. Nevertheless, whether HSP90AA1 coordinates DOX-induced ICD-related molecular changes and the accompanying remodeling of the breast tumor immune microenvironment remains insufficiently understood.

In the present study, we integrated bioinformatic analyses, clinical tissue validation, in vitro experiments, and an in vivo syngeneic breast cancer model to investigate the role of HSP90AA1 in breast cancer. We first analyzed ICD-related gene signatures and evaluated their associations with patient prognosis, immune cell infiltration, immune checkpoint expression, and human leukocyte antigen (HLA)-related molecules in publicly available breast cancer datasets. HSP90AA1 was subsequently identified as a candidate ICD-related regulator associated with unfavorable prognosis, and its aberrant expression was further validated in breast cancer tissues and cell lines. We then combined HSP90AA1 knockdown with DOX treatment to determine whether HSP90AA1 silencing enhances DOX-induced apoptosis and ICD-related molecular changes. Furthermore, using a syngeneic 4T1 breast cancer mouse model, we investigated whether HSP90AA1 knockdown combined with DOX suppresses tumor growth and alters immune infiltration-related markers within the tumor microenvironment. Collectively, our findings suggest that HSP90AA1 may function as a regulator of DOX responsiveness, ICD-related signaling, and immune microenvironment remodeling in breast cancer.

## 2. Materials and Methods

### 2.1. Data Acquisition and Processing

Breast cancer transcriptomic data and corresponding clinical information were obtained from The Cancer Genome Atlas Breast Invasive Carcinoma cohort (TCGA-BRCA). Samples with incomplete survival information were excluded from survival-related analyses. Gene expression data were normalized and processed before subsequent analyses. A set of immunogenic cell death (ICD)-related genes was collected from published literature and public gene set resources. The expression profiles of these ICD-related genes were extracted for differential expression analysis, protein–protein interaction analysis, consensus clustering, prognostic model construction, immune microenvironment analysis, and candidate gene validation.

### 2.2. Analysis of ICD-Related Genes and Molecular Subtypes

TCGA-BRCA gene-level FPKM expression profiles were used in this study. Duplicated gene symbols were aggregated using the avereps function in the limma package, and FPKM values were converted to TPM for subsequent analyses. The expression patterns of ICD-related genes between breast cancer and normal tissues were compared and visualized by heatmap. A protein–protein interaction network was constructed to explore the interactions among ICD-related genes. The optimal cluster number was determined according to the consensus clustering results. Patients were then classified into ICD-related subgroups. Kaplan–Meier survival analysis was performed to compare overall survival between ICD-related subgroups. Differentially expressed genes between ICD-related subgroups were identified and displayed using heatmap and volcano plot analyses.

### 2.3. Immune Microenvironment Analysis

The association between ICD-related subtypes and the tumor immune microenvironment was evaluated. ESTIMATE analysis was used to calculate the ESTIMATE score, ImmuneScore, StromalScore, and TumorPurity. The relative proportions of tumor-infiltrating immune cells were evaluated using immune cell deconvolution analysis. Differences in immune cell fractions between ICD-low and ICD-high groups were further compared. The expression levels of immune checkpoint-related genes and HLA-related genes were also compared between ICD-related subgroups to assess differences in immune regulation and antigen presentation-related features. Correlations among immune cell populations were analyzed to further characterize immune cell distribution patterns in the breast cancer microenvironment.

### 2.4. Construction and Evaluation of the ICD-Related Prognostic Model

Univariate Cox regression analysis was performed to identify ICD-related genes associated with overall survival. Candidate prognostic genes were then included in least absolute shrinkage and selection operator (LASSO) Cox regression analysis to construct an ICD-related prognostic signature. A risk score was calculated for each patient based on the expression levels of selected genes and their corresponding coefficients. Patients were divided into high-risk and low-risk groups according to the median risk score. The distribution of risk scores, survival status, and expression patterns of model genes were visualized. Kaplan–Meier survival analysis was used to compare overall survival between high-risk and low-risk groups.

Univariate and multivariate Cox regression analyses were further performed to evaluate whether the risk score was an independent prognostic factor when combined with clinical variables, including age, gender, T stage, N stage, and M stage. Correlations between the risk score and representative immune cell populations were analyzed to explore the relationship between the prognostic model and immune infiltration.

Among the genes included in the prognostic model, HSP90AA1 was selected for further validation. HSP90AA1 expression was compared between breast cancer tissues and normal breast tissues using public transcriptomic data. Paired tumor and matched normal tissue analysis was also performed when paired data were available. Kaplan–Meier survival analysis was used to compare overall survival between patients with high and low HSP90AA1 expression.

### 2.5. Clinical Tissue Samples and HSP90AA1 Validation

Paired breast cancer tissues and adjacent non-tumor tissues were collected from patients who underwent surgical resection at Nantong First People’s Hospital. A total of 28 paired breast cancer and adjacent non-tumor tissue samples were used specifically for validation of HSP90AA1 protein expression. All tissue samples were obtained from residual surgical specimens after routine pathological examination and were not collected through additional invasive procedures for research purposes. Fresh tissues were stored at −80 °C until protein extraction. This study was approved by the Ethics Committee of Nantong First People’s Hospital (Approval No. 2026-KT221). Written informed consent was waived by the ethics committee due to the retrospective use of residual surgical specimens.

### 2.6. Cell Culture and HSP90AA1 Expression Analysis

Human breast cancer cell lines BT-549, MDA-MB-231, and MCF-7, as well as the normal breast epithelial cell line MCF-10A, were obtained from the American Type Culture Collection (ATCC, Manassas, VA, USA). Cells were cultured in the appropriate medium supplemented with 10% fetal bovine serum and 1% penicillin–streptomycin at 37 °C in a humidified incubator containing 5% CO_2_.

HSP90AA1 protein expression in different cell lines was detected by Western blotting. HSP90AA1 mRNA expression was detected by quantitative real-time PCR. GAPDH was used as the internal control.

### 2.7. Quantitative Real-Time PCR

Total RNA was extracted from cells using TRIzol reagent (Takara Bio, Kusatsu, Shiga, Japan), and RNA concentration and purity were assessed spectrophotometrically. cDNA was synthesized using the PrimeScript RT kit, followed by quantitative real-time PCR using SYBR Green Real-time PCR Master Mix (both from Takara Bio) according to the manufacturer’s protocols. The thermal cycling conditions were 95 °C for 30 s, followed by 42 cycles of 95 °C for 10 s and 60 °C for 30 s, with subsequent melt-curve analysis to confirm amplification specificity. GAPDH was used as the internal reference gene, and relative mRNA expression was calculated using the 2^−ΔΔCt^ method.

The primer sequences were as follows: HSP90AA1 forward primer, 5′-TCTGCCTCTGGTGATGAGATGG-3′; HSP90AA1 reverse primer, 5′-CGTTCCACAAAGGCTGAGTTAGC-3′; GAPDH forward primer, 5′-CCTGGATACCGCAGCTAGGA-3′; and GAPDH reverse primer, 5′-GCGGCGCATACGAATGCCCC-3′.

### 2.8. siRNA Transfection and Doxorubicin Treatment

Small interfering RNA targeting HSP90AA1 and negative control siRNA were designed and synthesized by GenePharma (Suzhou, China). MDA-MB-231 cells were seeded into culture plates and transfected with si-HSP90AA1 or si-NC using Lipo8000 transfection reagent (Beyotime Biotechnology, Shanghai, China) according to the manufacturer’s instructions. After 48 h of transfection, HSP90AA1 knockdown efficiency was verified by Western blotting.

For doxorubicin treatment, cells were divided into four groups: si-NC, si-HSP90AA1, si-NC + DOX, and si-HSP90AA1 + DOX. Cells in the DOX-treated groups were treated with 1 μM doxorubicin for 24 h. After treatment, cells were collected for protein extraction and Western blot analysis.

### 2.9. Generation of Stable Hsp90aa1-Knockdown 4T1 Cells

*Hsp90aa1*-targeting shRNA lentivirus (sh-Hsp90aa1) and a non-targeting control lentivirus (sh-NC) were designed, constructed, and packaged by GenePharma (Suzhou, China). A U6 promoter-driven shRNA lentiviral backbone carrying a puromycin-resistance cassette was used, and the lentiviral vectors did not contain a GFP reporter. Stable 4T1-shHsp90aa1 and 4T1-shNC cell populations were generated by lentiviral transduction and subsequent selection by the supplier. The established stable cell populations were subsequently cultured and expanded in our laboratory. Hsp90aa1 knockdown efficiency was confirmed by Western blotting before the cells were used for animal experiments.

### 2.10. Western Blotting

Total protein was extracted from cells, clinical tissues, or mouse tumor tissues using RIPA lysis buffer supplemented with protease and phosphatase inhibitors (Beyotime Biotechnology, Shanghai, China), and concentrations were determined using a BCA assay (Beyotime Biotechnology, Shanghai, China). Equal amounts of protein (50 μg per lane) were separated by SDS-PAGE and transferred to PVDF membranes (Millipore, Burlington, MA, USA). After blocking with 5% non-fat milk or bovine serum albumin, membranes were incubated with primary antibodies overnight at 4 °C and subsequently with HRP-conjugated secondary antibodies. Protein bands were detected using enhanced chemiluminescence. Detailed antibody information, including working dilution factors, is provided in Supplementary Table S1. All WB original images are included in Supplementary File S1.

For clinical tissue validation, only HSP90AA1 protein expression was examined in paired breast cancer and adjacent non-tumor tissues. For the in vitro cell experiments, HSP90AA1, BAX, BCL-2, cleaved-caspase-3, CALR, HMGB1, eIF2α, p-eIF2α, CD8A, GZMB, FOXP3, and PD-L1 were examined. For animal tumor tissues, HSP90AA1, CALR, HMGB1, CD8A, GZMB, and PD-L1 were assessed.

### 2.11. Animal Experiments

Female BALB/c mice aged 6–8 weeks were purchased from the Laboratory Animal Center of Nantong University and maintained under specific pathogen-free conditions. All animal procedures were approved by the Animal Ethics Committee of Nantong University (Approval No. P20260618-001) and performed in accordance with institutional guidelines. Each experimental group contained four mice (*n* = 4).

To establish the syngeneic breast cancer model, 4T1-shNC or 4T1-shHsp90aa1 cells were suspended in sterile PBS and subcutaneously injected into BALB/c mice at 1 × 10^6^ cells per mouse. This inoculum was selected based on previously established 4T1 therapeutic tumor models using 1 × 10^6^ cells per mouse and was chosen to provide reproducible tumor establishment before treatment initiation on Day 7 [19]. Seven days after tumor cell inoculation, mice were treated with DOX or vehicle. Mice were divided into four groups: sh-NC, sh-Hsp90aa1, sh-NC + DOX, and sh- Hsp90aa1+ DOX. DOX was administered by intraperitoneal injection at 10 mg/kg on Days 7, 14, and 21. The 10 mg/kg dose was selected based on previously reported pharmacologically active DOX doses in 4T1 tumor-bearing BALB/c mouse models [20]. Control groups received the corresponding vehicle treatment.

On Day 28, mice were euthanized according to approved ethical procedures. Tumors were excised, photographed, weighed, and collected for protein extraction. Western blotting was performed to evaluate HSP90AA1 protein expression and the expression of ICD- and immune-associated proteins and the expression of ICD- and immune infiltration-related proteins, including CALR, HMGB1, CD8A, GZMB, and PD-L1.

### 2.12. Statistical Analysis

Statistical analyses were performed using GraphPad Prism (version 10.2.3) and R (version 4.3.2). Data are presented as mean ± standard deviation unless otherwise indicated. Paired tumor and adjacent non-tumor tissues were compared using appropriate paired tests, whereas two independent groups were analyzed using Student’s *t*-test or the Mann–Whitney U test according to data distribution. Multiple-group comparisons were performed by one-way ANOVA followed by appropriate post hoc tests. Survival was evaluated using Kaplan–Meier curves and the log-rank test, and prognostic factors were assessed by univariate and multivariate Cox regression analyses. Correlations between risk score and immune-cell infiltration were evaluated using correlation analyses. A two-sided *p* value < 0.05 was considered statistically significant.

## 3. Results

### 3.1. Identification of ICD-Related Genes and ICD-Associated Molecular Subtypes in Breast Cancer

**Figure 1:**
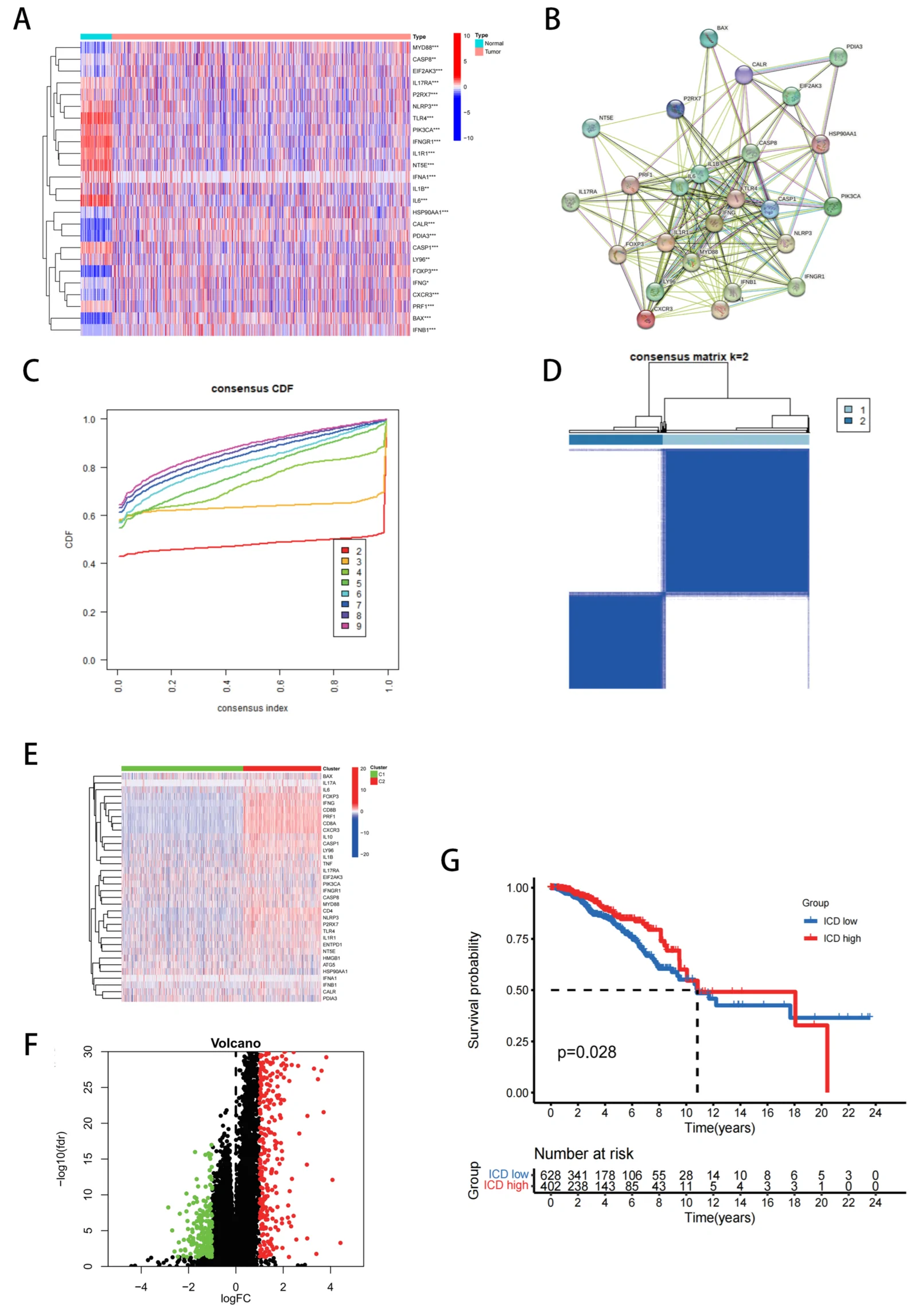
Identification of ICD-related genes and ICD-associated molecular subtypes in breast cancer. (**A**) Heatmap showing the expression patterns of immunogenic cell death (ICD)-related genes between breast cancer tissues and normal breast tissues in the TCGA-BRCA cohort. (**B**) Protein–protein interaction network of ICD-related genes. (**C**) Consensus cumulative distribution function (CDF) curves for different cluster numbers. (**D**) Consensus clustering matrix when k = 2, showing the classification of breast cancer samples into two ICD-related molecular subtypes. (**E**) Heatmap showing the expression profiles of ICD-related genes between the two ICD-related clusters. (**F**) Volcano plot showing differentially expressed genes between ICD-low and ICD-high subtypes. (**G**) Kaplan–Meier survival analysis comparing overall survival between ICD-low and ICD-high groups. * *p* < 0.05, ** *p* < 0.01, *** *p* < 0.001.

To explore the potential role of immunogenic cell death (ICD) in breast cancer, we first analyzed the expression profiles of ICD-related genes in breast cancer and normal breast tissues using TCGA-BRCA data. Differential expression analysis showed that multiple ICD-related genes were aberrantly expressed in breast cancer tissues compared with normal tissues, suggesting that ICD-related molecular alterations may be involved in breast cancer progression (Figure 1A). A protein–protein interaction network was then constructed to investigate the potential interactions among ICD-related molecules, which revealed a closely connected ICD-associated regulatory network (Figure 1B).

Based on the expression profiles of ICD-related genes, unsupervised consensus clustering was performed to classify breast cancer samples into distinct ICD-related molecular subtypes. The clustering results supported the classification of patients into ICD-low and ICD-high subgroups (Figure 1C,D). Kaplan–Meier survival analysis demonstrated that patients in different ICD-related subgroups had distinct overall survival outcomes, indicating that ICD-related molecular patterns were associated with breast cancer prognosis (Figure 1E). Furthermore, differential expression analysis between the ICD-low and ICD-high groups identified a set of ICD subtype-related differentially expressed genes, as shown by heatmap and volcano plot analyses (Figure 1F,G). The expression patterns of representative subtype-related differentially expressed genes are further shown in Figure S1.

### 3.2. ICD-Related Subtypes Are Associated with Distinct Immune Microenvironment Features

**Figure 2:**
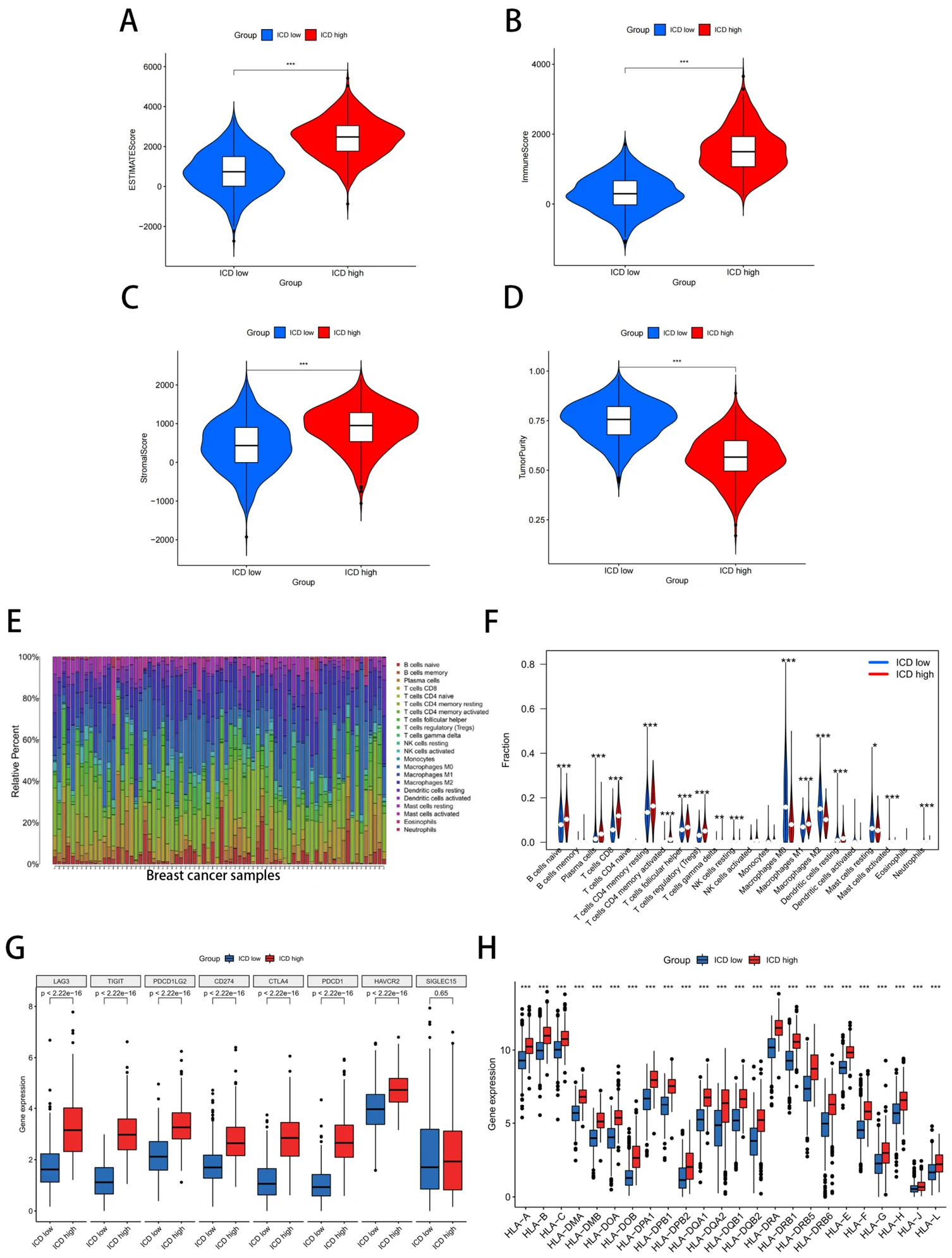
Immune microenvironment characteristics of ICD-low and ICD-high breast cancer subtypes. (**A**–**D**) Comparison of ESTIMATE score, ImmuneScore, StromalScore, and TumorPurity between ICD-low and ICD-high groups. (**E**) Stacked bar plot showing the relative proportions of 22 tumor-infiltrating immune cell types across breast cancer samples. (**F**) Comparison of immune cell fractions between ICD-low and ICD-high groups. (**G**) Expression levels of immune checkpoint-related genes, including LAG3, TIGIT, PDCD1LG2, CD274, CTLA4, PDCD1, HAVCR2, and SIGLEC15, between the two ICD subtypes. (**H**) Expression levels of HLA-related genes between ICD-low and ICD-high groups. ns, not significant; * *p* < 0.05, ** *p* < 0.01, *** *p* < 0.001.

To determine whether ICD-related molecular subtypes were associated with differences in the tumor immune microenvironment, we compared ESTIMATE-derived immune features between the ICD-low and ICD-high groups. The ICD-high group showed higher ESTIMATE score, ImmuneScore, and StromalScore, whereas TumorPurity was lower compared with the ICD-low group (Figure 2A–D). These results suggest that ICD-high tumors may be characterized by a more prominent immune and stromal component within the tumor microenvironment.

We next evaluated the relative proportions of tumor-infiltrating immune cells in breast cancer samples. The stacked bar plot showed heterogeneous immune cell compositions across individual breast cancer samples (Figure 2E). Further comparison between ICD-low and ICD-high groups revealed differences in the abundance of several immune cell populations, indicating that ICD-related subtypes were associated with distinct immune infiltration patterns (Figure 2F). In addition, multiple immune checkpoint-related genes were differentially expressed between the two ICD subtypes (Figure 2G). HLA-related genes also showed differential expression patterns between ICD-low and ICD-high tumors (Figure 2H), suggesting that ICD-related molecular features may be linked to immune regulation and antigen presentation in breast cancer.

To further characterize the immune landscape, correlation analysis was performed among the estimated immune cell populations. The correlation matrix showed complex positive and negative relationships among different tumor-infiltrating immune cell subsets, indicating coordinated immune cell distribution patterns within the breast cancer microenvironment (Figure S2).

### 3.3. Construction of an ICD-Related Prognostic Signature

**Figure 3:**
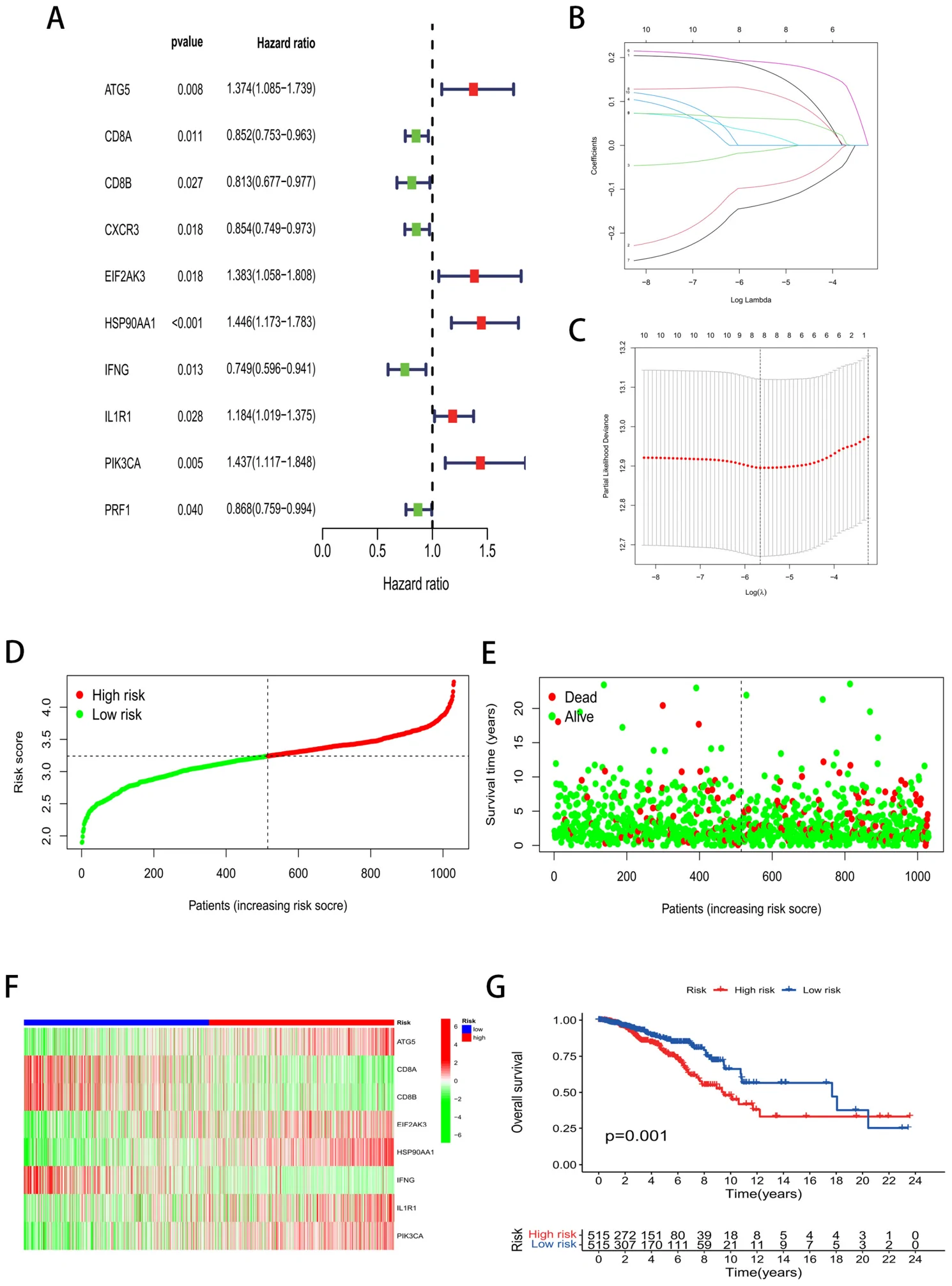
Construction of the ICD-related prognostic signature in breast cancer. (**A**) Forest plot showing ICD-related genes significantly associated with overall survival based on univariate Cox regression analysis. (**B**) LASSO coefficient profiles of candidate prognostic genes. (**C**) Cross-validation curve for selecting the optimal penalty parameter in the LASSO Cox regression model. (**D**) Distribution of risk scores in breast cancer patients ranked by increasing risk score. (**E**) Distribution of survival status according to increasing risk score. (**F**) Heatmap showing the expression patterns of prognostic signature genes between high-risk and low-risk groups. (**G**) Kaplan–Meier survival analysis comparing overall survival between high-risk and low-risk groups.

To evaluate the prognostic significance of ICD-related genes in breast cancer, univariate Cox regression was first used to identify genes associated with overall survival, followed by LASSO Cox regression to construct the prognostic signature, and an ICD-related prognostic signature was constructed (Figure 3A,B). The final risk model included representative ICD-related genes, including ATG5, CD8A, CD8B, CXCR3, EIF2AK3, HSP90AA1, IFNG, IL1R1, PIK3CA, and PRF1.

A risk score was calculated for each patient from the expression levels and corresponding coefficients of the model genes, and patients were stratified into high- and low-risk groups using the median score as the cutoff. The distribution of risk scores and survival status showed that patients in the high-risk group tended to have poorer survival outcomes than those in the low-risk group (Figure 3C,D). The heatmap showed distinct expression patterns of model genes between the high-risk and low-risk groups (Figure 3E). Kaplan–Meier survival analysis further confirmed that the high-risk group had significantly worse overall survival compared with the low-risk group (Figure 3F,G).

### 3.4. Prognostic Value of the ICD-Related Risk Model and Validation of HSP90AA1 Expression in Breast Cancer Tissues

**Figure 4:**
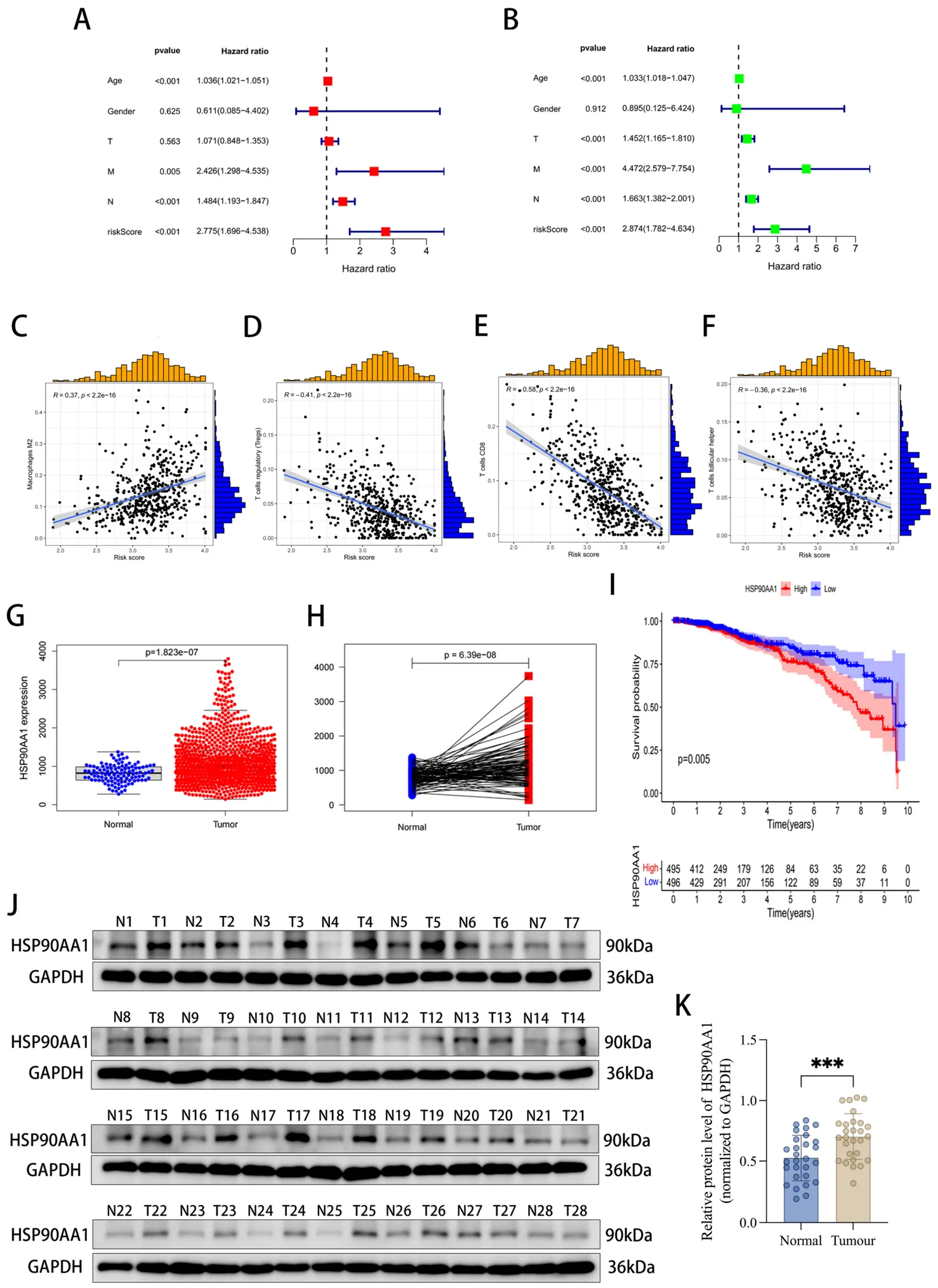
Prognostic value of the ICD-related risk model and validation of HSP90AA1 expression in breast cancer. (**A**) Univariate Cox regression analysis evaluating the prognostic value of the ICD-related risk score and clinicopathological variables, including age, gender, T stage, M stage, and N stage. (**B**) Multivariate Cox regression analysis assessing the independent prognostic value of the risk score after adjustment for clinicopathological variables. (**C**–**F**) Correlation analyses between the ICD-related risk score and representative tumor-infiltrating immune cell populations, including macrophages M2, regulatory T cells, CD8 T cells, and follicular helper T cells. (**G**) HSP90AA1 expression in normal breast tissues and breast cancer tissues based on public transcriptomic data. (**H**) Paired comparison of HSP90AA1 expression between breast cancer tissues and matched normal tissues. (**I**) Kaplan–Meier survival analysis comparing overall survival between patients with high and low HSP90AA1 expression. (**J**) Western blot analysis of HSP90AA1 protein expression in 28 paired breast cancer tissues and adjacent non-tumor tissues. N, adjacent non-tumor tissue; T, tumor tissue. (**K**) Quantification of HSP90AA1 protein expression in paired clinical tissue samples. GAPDH was used as the internal loading control. Data are presented as mean ± SD. *** *p* < 0.001.

To assess the independent prognostic value of the ICD-related risk score, univariate and multivariate Cox regression analyses were performed with age, gender, and TNM stage as covariates. The results indicated that the risk score was closely associated with overall survival and may provide additional prognostic information beyond conventional clinicopathological parameters (Figure 4A,B).

We further explored the relationship between the ICD-related risk score and immune cell infiltration. Correlation analyses showed that the risk score was associated with several representative tumor-infiltrating immune cell populations, including macrophages M2, regulatory T cells, CD8 T cells, and follicular helper T cells, suggesting a potential connection between the ICD-related prognostic model and the immune microenvironment (Figure 4C–F).

Among the genes included in the prognostic model, HSP90AA1 was selected for further validation because of its elevated expression in breast cancer and its potential role in tumor progression and therapeutic resistance. Public transcriptomic dataset analysis showed that HSP90AA1 expression was significantly higher in breast cancer tissues than in normal breast tissues (Figure 4G). Consistently, paired sample analysis also demonstrated increased HSP90AA1 expression in breast cancer tissues compared with matched normal tissues (Figure 4H). Kaplan–Meier survival analysis further showed that patients with high HSP90AA1 expression had worse overall survival than those with low HSP90AA1 expression (Figure 4I).

To validate this finding experimentally, HSP90AA1 protein expression was examined in 28 paired breast cancer and adjacent non-tumor tissues. Western blot analysis showed that HSP90AA1 protein expression was higher in breast cancer tissues than in paired adjacent tissues (Figure 4J,K). These results support HSP90AA1 as a candidate ICD-related regulator with prognostic relevance in breast cancer.

### 3.5. HSP90AA1 Knockdown Enhances DOX-Induced Apoptotic and ICD-Related Molecular Changes in Breast Cancer Cells

**Figure 5:**
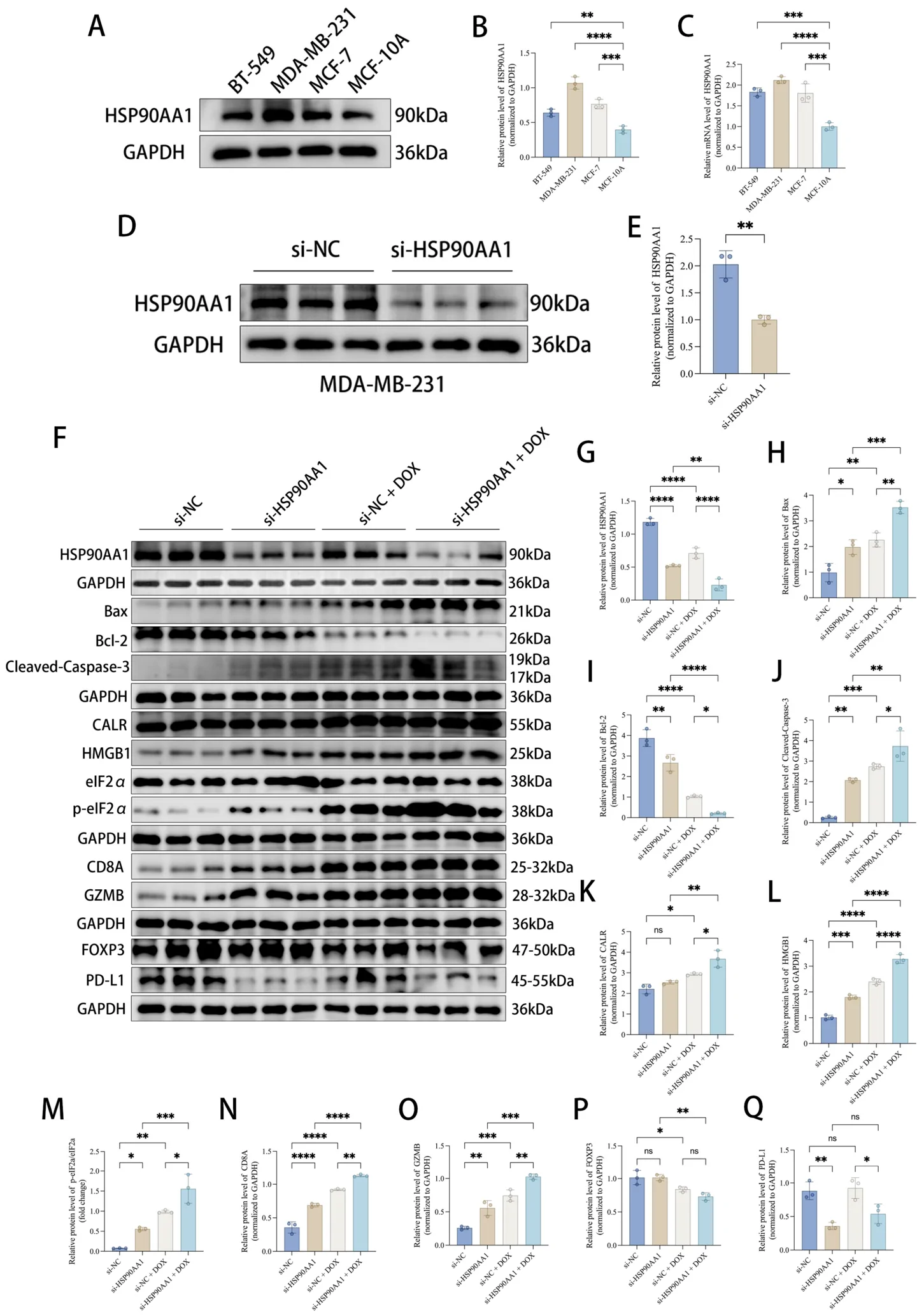
*HSP90AA1* knockdown enhances DOX-induced apoptotic and ICD-related molecular changes in MDA-MB-231 cells. (**A**) Western blot analysis of HSP90AA1 protein expression in breast cancer cell lines BT-549, MDA-MB-231, and MCF-7, and the normal breast epithelial cell line MCF-10A. (**B**) Quantification of HSP90AA1 protein expression in different cell lines. (**C**) qRT-PCR analysis of HSP90AA1 mRNA expression in different cell lines. (**D**) Western blot analysis confirming HSP90AA1 knockdown efficiency in MDA-MB-231 cells transfected with si-HSP90AA1 or si-NC. (**E**) Quantification of HSP90AA1 protein expression after siRNA-mediated knockdown. (**F**) Western blot analysis of HSP90AA1, apoptosis-related proteins, ICD-related proteins, and immune-associated markers in MDA-MB-231 cells from the si-NC, si-HSP90AA1, si-NC + DOX, and si-HSP90AA1 + DOX groups. (**G**–**Q**) Quantification of HSP90AA1, BAX, BCL-2, cleaved-caspase-3, CALR, HMGB1, p-eIF2α/eIF2α, CD8A, GZMB, FOXP3, and PD-L1 protein levels, respectively. GAPDH was used as the internal loading control. Data are presented as mean ± SD. ns, not significant; * *p* < 0.05, ** *p* < 0.01, *** *p* < 0.001, **** *p* < 0.0001.

To further characterize HSP90AA1 expression in breast cancer cells, we first examined HSP90AA1 expression in human breast cancer cell lines and the normal breast epithelial cell line MCF-10A. Compared with MCF-10A cells, breast cancer cell lines showed increased HSP90AA1 expression at both protein and mRNA levels (Figure 5A–C). Based on these findings, MDA-MB-231 cells were selected for subsequent knockdown and doxorubicin treatment experiments.

To investigate the functional role of HSP90AA1 in breast cancer cells, HSP90AA1 was knocked down in MDA-MB-231 cells using siRNA. Western blotting confirmed that HSP90AA1 expression was effectively reduced after si-HSP90AA1 transfection compared with the si-NC group (Figure 5D,E).

We then examined whether HSP90AA1 knockdown influenced the response of breast cancer cells to doxorubicin (DOX). Cells were divided into four groups: si-NC, si-HSP90AA1, si-NC + DOX, and si-HSP90AA1 + DOX. Western blot analysis showed that DOX treatment increased the expression of pro-apoptotic proteins, including BAX and cleaved-caspase-3, and decreased the expression of the anti-apoptotic protein BCL-2. Notably, these DOX-induced apoptotic changes were further enhanced by HSP90AA1 knockdown (Figure 5F–I). These results suggest that HSP90AA1 knockdown may increase DOX-induced apoptotic responses in breast cancer cells.

Because DOX is known to induce ICD-related molecular events, we next examined ICD-associated markers. DOX treatment increased the expression of ICD-related proteins, including CALR and HMGB1, and enhanced eIF2α phosphorylation. HSP90AA1 knockdown further strengthened these ICD-related molecular changes under DOX treatment (Figure 5J–M). In addition, the expression levels of immune-associated proteins, including CD8A, GZMB, FOXP3, and PD-L1, were also altered after HSP90AA1 knockdown and DOX treatment, suggesting that HSP90AA1 may participate in the regulation of chemotherapy-associated immune-related signaling in breast cancer cells (Figure 5N–Q). Together, these findings indicate that HSP90AA1 knockdown enhances DOX-induced apoptotic responses and ICD-related molecular changes in vitro.

### 3.6. Hsp90aa1 Knockdown Enhances the Antitumor Effect of DOX in a 4T1 Syngeneic Breast Cancer Model

**Figure 6:**
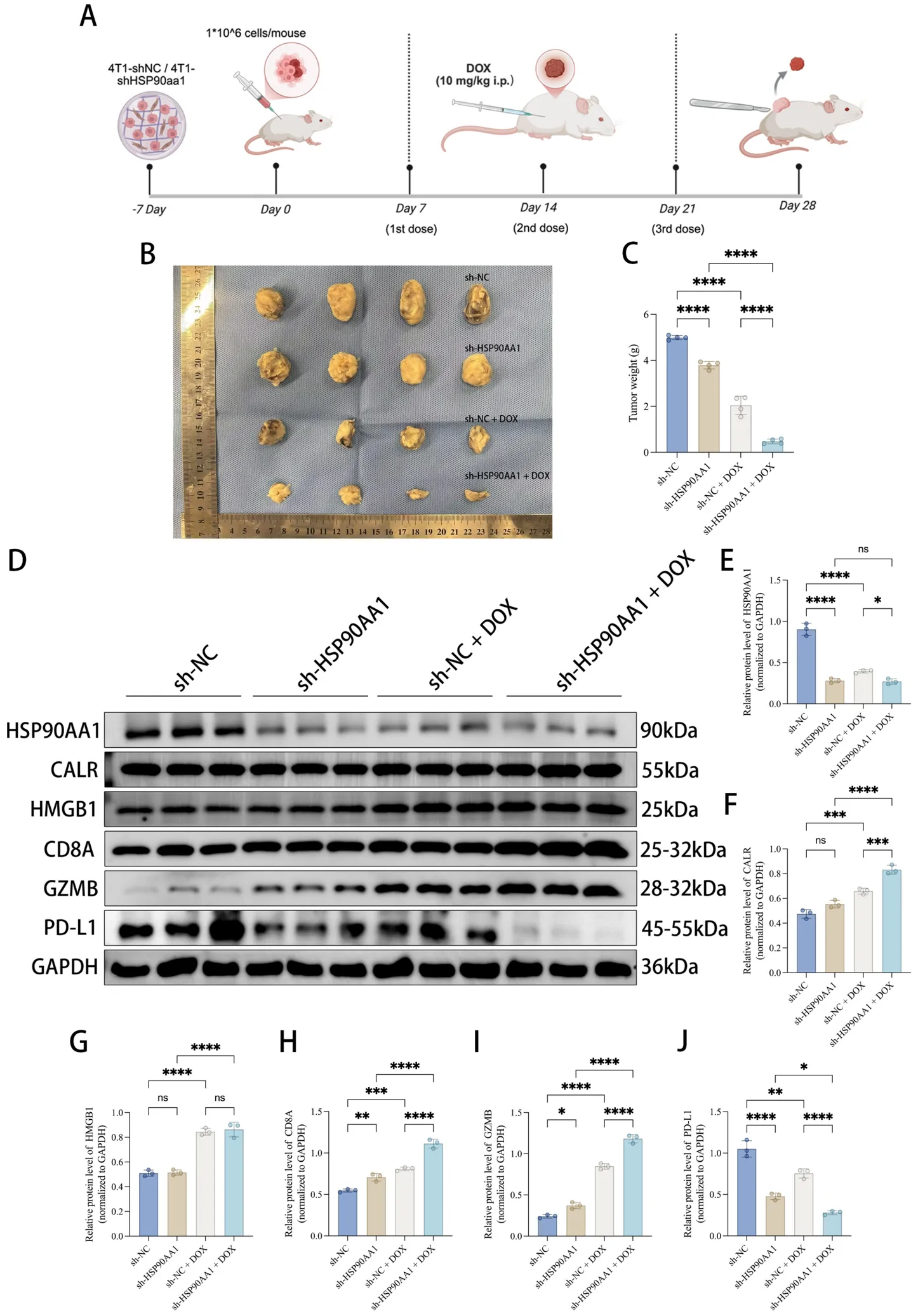
*Hsp90aa1* knockdown enhances the antitumor effect of DOX in a 4T1 syngeneic breast cancer mouse model. (**A**) Schematic illustration of the in vivo experimental design. 4T1-shNC or 4T1-shHsp90aa1 cells were subcutaneously injected into BALB/c mice, followed by DOX treatment on Days 7, 14, and 21. Tumors were collected on Day 28. (**B**) Representative images of excised tumors from the sh-NC, sh-Hsp90aa1, sh-NC + DOX, and sh-Hsp90aa1 + DOX groups. (**C**) Tumor weight in the four treatment groups. (**D**) Western blot analysis of HSP90AA1, CALR, HMGB1, CD8A, GZMB, and PD-L1 protein expression in excised tumor tissues. (**E**–**J**) Quantification of HSP90AA1, CALR, HMGB1, CD8A, GZMB, and PD-L1 protein levels, respectively. GAPDH was used as the loading control. Data are presented as mean ± SD. ns, not significant; * *p* < 0.05, ** *p* < 0.01, *** *p* < 0.001, **** *p* < 0.0001.

Before performing the in vivo experiments, we further verified the stability of Hsp90aa1 knockdown in cultured 4T1 cells. Western blot analysis showed that HSP90AA1 protein expression was markedly reduced in 4T1-shHsp90aa1 cells compared with 4T1-shNC cells (Figure S3A,B), confirming stable Hsp90aa1 knockdown in vitro. These validated stable cell populations were subsequently used for establishment of the syngeneic breast cancer model. To further validate the role of Hsp90aa1 in vivo, a 4T1 syngeneic breast cancer mouse model was established using 4T1-shNC or 4T1-shHsp90aa1 cells. After tumor establishment, mice were treated with vehicle or DOX according to the experimental schedule (Figure 6A). Representative tumor images and terminal tumor weights showed that both Hsp90aa1 knockdown and DOX treatment reduced terminal tumor burden, whereas the combination of Hsp90aa1 knockdown and DOX produced the greatest reduction in tumor burden (Figure 6B,C).

Western blot analysis confirmed that HSP90AA1 protein expression was reduced in tumors derived from 4T1-shHsp90aa1 cells compared with 4T1-shNC tumors (Figure 6D,E). We next examined ICD- and immune infiltration-related proteins in tumor tissues. The expression levels of CALR and HMGB1 increased after DOX treatment and were further enhanced in the sh-Hsp90aa1 + DOX group (Figure 6F,G). In addition, tumors in the combined treatment group showed increased expression of CD8A and GZMB, consistent with increased expression of molecular markers associated with cytotoxic immune-cell infiltration or activity, rather than providing direct quantitative characterization of immune-cell populations (Figure 6H,I). PD-L1 expression also changed among the treatment groups, indicating that *Hsp90aa1* knockdown and DOX treatment may influence immune regulatory signaling in vivo (Figure 6J).

Overall, these in vivo results suggest that *Hsp90aa1* knockdown enhances the antitumor efficacy of DOX and is associated with increased ICD-related molecular changes and antitumor immune infiltration-related markers in breast cancer.

## 4. Discussion

Doxorubicin is widely used in breast cancer treatment, but therapeutic resistance and treatment-associated immune escape remain major obstacles limiting its long-term clinical benefit [21,22]. Immunogenic cell death (ICD) provides an important mechanistic link between chemotherapy-induced tumor cell death and the activation of antitumor immunity [23]. However, the molecular regulators that coordinate doxorubicin response, ICD-related signaling, and immune microenvironment remodeling in breast cancer remain incompletely understood. In the present study, we integrated bioinformatic analyses with clinical tissue validation, in vitro experiments, and an in vivo syngeneic breast cancer model to investigate the role of HSP90AA1. Our results showed that ICD-related gene expression patterns were associated with patient prognosis, immune cell infiltration, immune checkpoint expression, and human leukocyte antigen (HLA)-related antigen-presentation features. HSP90AA1 was identified as a candidate gene associated with ICD-related molecular features, was highly expressed in breast cancer, and was associated with unfavorable prognosis. Functionally, HSP90AA1 knockdown enhanced doxorubicin-induced apoptosis and ICD-related molecular changes in breast cancer cells. In vivo, HSP90AA1 knockdown enhanced the antitumor effect of doxorubicin and was accompanied by increased expression of ICD-related and immune infiltration/activity-associated markers.

ICD is increasingly recognized as an important mechanism through which selected chemotherapeutic agents convert tumor cell death into adaptive antitumor immunity [24,25]. During ICD, dying tumor cells expose calreticulin (CALR) on the cell surface and release damage-associated molecular patterns, including high-mobility group box 1 (HMGB1) and extracellular ATP, which promote dendritic cell-mediated uptake of tumor antigens, antigen presentation, and the activation of cytotoxic T-cell responses [24,26]. Doxorubicin is considered a prototypical ICD-inducing chemotherapeutic agent and has been shown to induce ICD-associated molecular and immune responses in breast cancer models [24,26]. In the present study, ICD-related genes were differentially expressed between breast cancer and normal tissues, and the identified ICD-related molecular subtypes exhibited distinct survival outcomes. A heatmap of subtype-associated differentially expressed genes further demonstrated the transcriptional heterogeneity between ICD-low and ICD-high tumors. Collectively, these findings suggest that ICD-related molecular alterations may define biologically distinct breast cancer states with potential prognostic relevance.

The immune microenvironment is a key determinant of breast cancer progression and therapeutic response [27,28]. In our analysis, the ICD-high group exhibited higher ESTIMATE, Immune, and Stromal Scores, together with lower tumor purity, suggesting a greater abundance of non-malignant immune and stromal components in ICD-high tumors. Immune cell deconvolution further revealed distinct immune infiltration patterns between the ICD-low and ICD-high subtypes. In addition, immune checkpoint-related and human leukocyte antigen (HLA)-related genes were differentially expressed between the two ICD subtypes, suggesting that ICD-related molecular features may be closely associated with immune regulation and antigen-presentation programs. The correlation matrix of 22 tumor-infiltrating immune cell types revealed complex positive and negative associations among different immune cell subsets, further supporting the coordinated organization of immune populations within the breast cancer microenvironment [28,29].

To further evaluate the prognostic value of ICD-related genes, we constructed an ICD-related risk model comprising ATG5, CD8A, CD8B, CXCR3, EIF2AK3, HSP90AA1, IFNG, IL1R1, PIK3CA, and PRF1. Collectively, these genes span multiple biological programs relevant to tumor immunity and treatment response. ATG5 and EIF2AK3 are involved in autophagy and endoplasmic reticulum stress-related signaling, respectively [30]. CD8A, CD8B, CXCR3, IFNG, and PRF1 are associated with T-cell infiltration, trafficking, interferon-mediated responses, and cytotoxic immune activity, whereas IL1R1 and PIK3CA participate in inflammatory signaling and the regulation of the breast tumor immune microenvironment [31,32,33]. Patients in the high-risk group exhibited significantly poorer survival outcomes than those in the low-risk group, and the risk score was associated with distinct immune infiltration features. Collectively, these findings suggest that ICD-related transcriptional patterns may have both prognostic and immunological significance in breast cancer.

Importantly, computational prioritization should be distinguished from experimental demonstration of therapeutic validity. Bioinformatic, proteomic, genetic, and drug–target prediction approaches can efficiently identify candidate molecules and generate mechanistic hypotheses, but such associations do not by themselves establish causality or clinical actionability. Recent studies have illustrated how proteomic Mendelian randomization and integrative genetic analyses can prioritize potential therapeutic targets in breast cancer, while computational drug–target interaction models can further facilitate candidate identification [34]. Nevertheless, predictions generated by these approaches require independent experimental and ultimately clinical validation. Accordingly, in the present study, the bioinformatic identification of HSP90AA1 was considered a candidate-prioritization step rather than evidence of a causal therapeutic target, and its biological relevance was subsequently evaluated using clinical tissues, gene-silencing experiments, and an in vivo breast cancer model.

Among the genes included in the prognostic model, HSP90AA1 was selected for further investigation. HSP90AA1 encodes HSP90α, a stress-inducible cytosolic molecular chaperone that participates in protein folding and maintains the stability and activity of multiple oncogenic client proteins [35]. Aberrant HSP90AA1 expression may enhance the capacity of tumor cells to adapt to proteotoxic and treatment-induced stress, thereby promoting tumor cell survival and therapeutic resistance. Indeed, chemotherapy-induced HSP90AA1 expression has been shown to facilitate autophagy-associated drug resistance in tumor cells [36]. Consistent with these biological functions, our public transcriptomic analyses showed that HSP90AA1 expression was significantly increased in breast cancer tissues compared with normal tissues in both unpaired and paired sample analyses. High HSP90AA1 expression was also associated with unfavorable overall survival. Furthermore, Western blot analysis of 28 paired clinical samples confirmed that HSP90AA1 protein expression was higher in breast cancer tissues than in paired adjacent non-tumor tissues. These clinical specimens were used primarily for expression validation and were not designed to provide quantitative histopathological assessment of apoptosis, necrosis, or immune-cell infiltration. HSP90AA1 expression was also elevated in breast cancer cell lines compared with normal breast epithelial cells at both the protein and mRNA levels. These observations are consistent with a previous large-scale breast cancer study showing that elevated HSP90AA1 mRNA and protein expression was associated with aggressive clinicopathological features and shorter breast cancer-specific survival [37]. Collectively, these findings support the clinical and biological relevance of HSP90AA1 in breast cancer.

An additional mechanism through which HSP90AA1 may influence treatment response involves tumor metabolic adaptation. The HSP90 chaperone network is closely linked to mitochondrial function, cellular bioenergetics, and adaptation to metabolic stress. Of particular relevance to breast cancer, HSP90AA1 has recently been shown to interact with the mitochondrial outer-membrane protein VDAC1 and contribute to DOX resistance through PI3K/AKT-related signaling, whereas *HSP90AA1* silencing restored DOX sensitivity in resistant breast cancer cells [16]. In addition, TRAP1, a mitochondrial member of the HSP90 family, has been reported to maintain oxidative phosphorylation and ATP production and thereby promote DOX resistance in breast cancer cells [38]. These findings raise the possibility that *HSP90AA1* depletion may reduce the metabolic and stress-adaptive capacity of breast cancer cells, thereby increasing their susceptibility to DOX-induced stress. However, metabolic phenotypes were not directly assessed in the present study. Therefore, whether metabolic remodeling mechanistically contributes to the enhanced DOX response following HSP90AA1 depletion remains to be determined.

Functionally, HSP90AA1 knockdown enhanced DOX-induced apoptosis in MDA-MB-231 cells. Compared with DOX treatment alone, the combination of HSP90AA1 knockdown and DOX further increased the expression of BAX and cleaved caspase-3 while decreasing the expression of BCL-2. These findings suggest that HSP90AA1 may exert a cytoprotective effect against DOX-induced apoptotic stress. Consistent with the broader involvement of the HSP90 chaperone network in treatment response, pharmacological inhibition of HSP90 has been shown to promote apoptosis through alterations in BAX/BCL-2-related mitochondrial apoptotic signaling and activation of caspase-dependent cell death [39,40]. Moreover, inhibition of heat shock proteins has been reported to resensitize DOX-resistant breast cancer cells to DOX and enhance apoptosis [41]. Therefore, targeting HSP90AA1 may increase the susceptibility of breast cancer cells to DOX-induced cell death. More broadly, strategies aimed at improving anthracycline-based breast cancer therapy are being actively explored. For example, Yuan et al. developed reduction-responsive lipophilic epirubicin prodrug nanoassemblies that improved antitumor efficacy while reducing treatment-related toxicity, illustrating how optimization of anthracycline delivery may further enhance therapeutic performance in breast cancer.

The mechanistic relationship between HSP90AA1 depletion and ICD-related signaling requires cautious interpretation. Our findings do not establish HSP90AA1 as a canonical upstream initiator of ICD. Rather, we propose that HSP90AA1 primarily functions as a stress-buffering molecular chaperone that supports proteostasis and cellular adaptation to treatment-induced stress. Accordingly, HSP90AA1 depletion may reduce the capacity of breast cancer cells to tolerate DOX-induced proteotoxic, oxidative, and endoplasmic-reticulum stress, thereby lowering the threshold for cell death and amplifying molecular events associated with ICD. Consistent with this stress-amplification model, *HSP90AA1* knockdown enhanced DOX-induced apoptotic signaling, CALR and HMGB1 abundance, and eIF2α phosphorylation in our experiments. Chaperone-dependent protein homeostasis is closely interconnected with cellular metabolic and stress adaptation in breast cancer [42]. For example, Zhou et al. demonstrated that chaperone-mediated autophagy promoted metabolic reprogramming and breast cancer progression through degradation of citrate synthase and fumarate hydratase. Calcium-dependent signaling may represent an additional determinant of treatment response, as perturbation of intracellular Ca^2+^ homeostasis can induce mitochondrial stress and disrupt metabolic homeostasis in breast cancer cells [43]. Recent evidence further indicates that tumor-promoting metabolic remodeling can influence cancer progression and chemosensitivity, suggesting that metabolic context may modify the response to HSP90AA1 depletion and DOX treatment [44]. Although these studies do not directly establish a role for HSP90AA1 in ICD, they support the broader concept that disruption of chaperone-dependent adaptive programs and associated metabolic stress responses can influence the ability of breast cancer cells to withstand treatment-induced stress. HSP90AA1-dependent stress adaptation is therefore likely to operate within a complex network of metabolic, oxidative, inflammatory, immune, and systemic stress signals rather than through a strictly linear pathway. Huang et al. highlighted such multisystem stress–cancer interactions, providing a broader biological context for interpreting the HSP90AA1-associated stress response observed here [45]. Therefore, HSP90AA1 is best interpreted in the present study as a potential modulator of DOX-associated cellular stress and ICD-related molecular changes rather than as a demonstrated upstream regulator of ICD.

Importantly, enhanced cytotoxicity and specific regulated cell-death mechanisms should be distinguished from bona fide ICD. Apoptosis, ferroptosis, pyroptosis, and other regulated cell-death programs are defined by distinct molecular execution mechanisms, whereas ICD refers to the capacity of dying cells to generate sufficient antigenic and adjuvant signals to elicit adaptive antitumor immunity. These concepts are therefore related but not interchangeable. In the present study, increased BAX and cleaved caspase-3 together with decreased BCL-2 support enhanced apoptotic signaling following HSP90AA1 knockdown and DOX treatment. By contrast, ferroptosis-specific events, including iron-dependent lipid peroxidation and GPX4/SLC7A11-related regulation, and pyroptosis-associated events, including caspase activation and gasdermin cleavage, were not examined. Recent studies further illustrate that these alternative cell-death programs can independently influence therapeutic responses. Zhou et al. showed that induction of ferroptosis contributed to the antitumor activity of chrysotoxine in cervical cancer [46,47], whereas Li et al. demonstrated that pharmacologically induced pyroptosis could reshape the tumor microenvironment and enhance anti-PD-1 therapy in melanoma [48]. These studies provide examples of the mechanistic diversity and therapeutic relevance of regulated tumor-cell death but do not establish the involvement of ferroptosis or pyroptosis in our *HSP90AA1*-knockdown model. Accordingly, our findings are interpreted as enhanced apoptotic responses accompanied by ICD-related molecular changes, while the potential contribution of other regulated cell-death pathways remains to be determined.

PD-L1 is a key immune checkpoint ligand that contributes to tumor immune escape by suppressing antitumor T-cell responses. In triple-negative breast cancer, tumor-intrinsic PD-L1 signaling has also been linked to cancer cell plasticity, treatment resistance, and metastatic progression [49]. In our cell and animal experiments, PD-L1 abundance was altered following Hsp90aa1 knockdown and DOX treatment, suggesting that HSP90AA1/Hsp90aa1 may participate in chemotherapy-associated immune regulatory signaling. This possibility is biologically plausible because HSP90 inhibition has been shown to reduce tumor-cell surface PD-L1 expression and increase activated CD8-positive T-cell infiltration in preclinical tumor models [50]. Moreover, HSP90 can stabilize STAT1 and thereby support IFNγ-induced PD-L1 expression, providing a potential link between HSP90 activity and adaptive immune resistance [51]. A chemotherapy-induced HSF1–HSP90 axis has also been reported to increase functional cell-surface PD-L1 and attenuate cytotoxic T-cell responses, although this mechanism was demonstrated in a non-breast cancer model [52]. Nevertheless, PD-L1 expression is regulated by multiple and context-dependent mechanisms, including tumor-intrinsic oncogenic signaling, chemotherapy-induced stress responses, inflammatory stimulation, and adaptive immune feedback. Therefore, the precise mechanism through which *HSP90AA1*/*Hsp90aa1* knockdown alters PD-L1 expression in DOX-treated breast cancer requires further investigation. Importantly, these findings do not establish direct regulation of immune-related genes by HSP90AA1/Hsp90aa1. HSP90AA1/Hsp90aa1 depletion may instead reduce cellular stress tolerance and secondarily alter stress-responsive immune-regulatory transcription following DOX treatment. Because temporal signaling, transcriptional regulation, and rescue experiments were not performed, the observed immune-associated changes should therefore be considered HSP90AA1/Hsp90aa1-dependent associations rather than evidence of a direct molecular mechanism. In addition, changes in immune-associated proteins measured in cultured tumor cells should not be interpreted as evidence of immune cell infiltration, because immune infiltration cannot be directly evaluated in a tumor-cell monoculture system. Collectively, these findings suggest a potential link among HSP90AA1/Hsp90aa1, chemotherapy response, and immune checkpoint-related signaling in breast cancer.

From a translational perspective, these observations also raise the possibility that HSP90AA1-targeted strategies could complement existing cancer immunotherapies. Monoclonal antibody-based immunotherapies, particularly immune checkpoint inhibitors targeting PD-1/PD-L1 or CTLA-4, have become important components of modern cancer treatment, although their efficacy varies substantially among patients and tumor types [53]. The composition and functional state of the tumor microenvironment are increasingly recognized as major determinants of immunotherapy efficacy [54]. Although Wang et al. focused on ovarian cancer, the broader principle that immunosuppressive and metabolically altered tumor microenvironments can influence immunotherapeutic responses may also be relevant to other solid tumors. Given the reported effects of HSP90 inhibition on PD-L1-associated signaling and antitumor immune responses, HSP90AA1 targeting may potentially enhance sensitivity to immune checkpoint-based therapy by modifying tumor-intrinsic stress adaptation and immune-regulatory features of the tumor microenvironment. However, this possibility remains speculative in the context of the present study because HSP90AA1-targeted intervention was not directly combined with immune checkpoint blockade. Future studies using immunocompetent models will be required to determine whether HSP90AA1-targeted strategies can enhance the therapeutic efficacy of anti-PD-1/PD-L1 or other immunotherapeutic approaches.

The in vivo results further supported the role of Hsp90aa1 in modulating the response to doxorubicin and immune-related tumor changes. In the 4T1 syngeneic breast cancer model, both Hsp90aa1 knockdown and doxorubicin treatment reduced terminal tumor burden, whereas their combination produced the greatest reduction. Importantly, enhanced DOX sensitivity in experimental models should not be assumed to translate directly into improved clinical therapeutic efficacy. Although Hsp90aa1 knockdown enhanced the antitumor response to DOX in our syngeneic model, clinically relevant efficacy will also depend on pharmacokinetics, intratumoral drug penetration, stromal interactions, and systemic toxicity. Therefore, the present findings should be interpreted as preclinical evidence of sensitization rather than validation of a clinically effective HSP90AA1-targeted strategy. Tumor tissues from the combined treatment group showed increased CALR and HMGB1 expression, consistent with enhanced ICD-related molecular changes in vivo. In addition, increased CD8A and GZMB levels were consistent with molecular changes associated with cytotoxic immune-cell infiltration or activity [55]. However, these immune-related findings should be interpreted cautiously. Although the bioinformatic analyses identified differences in inferred immune-cell populations between ICD-related subgroups, such computational estimates do not directly demonstrate changes in immune-cell function. Similarly, increased CD8A and GZMB levels in whole-tumor lysates do not provide direct quantitative information regarding immune-cell abundance, phenotype, spatial distribution, or functional state. Changes in PD-L1 expression likewise reflect immune-regulatory signaling rather than direct evidence of immune activation. Therefore, the proposed Hsp90aa1–DOX–immune relationship in the mouse model should be interpreted as an association with immune-related tumor microenvironment remodeling rather than definitive evidence of functional antitumor immune activation. Further studies using multiparameter flow cytometry, immunohistochemistry or immunofluorescence, and functional immune-cell assays will be required to validate these findings.

Spatial heterogeneity represents an additional consideration when interpreting the immune-related tumor microenvironment changes observed in this study. Computational immune-cell deconvolution and whole-tumor protein measurements provide population-level or average molecular information but do not preserve the spatial organization of immune cells within distinct tumor regions. Importantly, immunostimulatory and immunosuppressive populations may coexist in spatially distinct niches, and their biological effects can depend on their proximity to malignant cells and other immune or stromal populations. Li et al. recently demonstrated in another tumor context that MDSCs undergo marked spatial redistribution during tumor progression and that their regional localization is associated with immune suppression and therapeutic resistance [56]. More broadly, spatially resolved profiling can reveal intratumoral heterogeneity and local tumor–immune–stromal interactions that may be obscured by conventional bulk or static measurements [57]. Therefore, the increased CD8A and GZMB levels observed in the present study should not be interpreted as evidence of spatially uniform antitumor immune activation. Future studies incorporating spatial transcriptomics, multiplex immunofluorescence, or spatial proteomic approaches will be required to determine whether Hsp90aa1 depletion and DOX treatment are associated with changes in specific immune-active or immunosuppressive niches within the breast tumor microenvironment.

The potential therapeutic relevance of HSP90AA1 targeting should also be considered in the context of breast cancer heterogeneity. The present functional experiments were performed in selected breast cancer models and therefore do not establish that HSP90AA1-targeted strategies would be equally effective across all molecular subtypes. Differences in oncogenic signaling, dependence on HSP90AA1, immune microenvironment characteristics, and sensitivity to anthracycline-based chemotherapy may influence treatment responses. Accordingly, our findings support HSP90AA1 targeting as a potential therapeutic strategy rather than establishing HSP90AA1 as a universally applicable target across all breast cancer subtypes. Future studies incorporating subtype-stratified cell models, animal models, and clinical cohorts will be required to determine which patient populations may be most likely to benefit from HSP90AA1-targeted strategies.

An important consideration for the therapeutic translation of HSP90AA1 targeting is its potential safety and therapeutic window. HSP90 proteins are essential components of cellular protein homeostasis, and systemic or sustained pharmacological inhibition may therefore affect normal tissues in addition to tumor cells. Indeed, the clinical development of several HSP90 inhibitors has been limited by dose-dependent toxicities, highlighting the need to distinguish antitumor activity from acceptable systemic exposure. Consequently, HSP90AA1-targeted strategies should not be assumed to possess intrinsic tumor selectivity. Approaches that preferentially restrict HSP90-targeted intervention to tumor tissues may help improve the therapeutic window [58]. Tumor-directed delivery systems and targeted formulations have shown the potential to enhance tumor accumulation and reduce systemic exposure in preclinical settings, while more selective HSP90 modulators may further limit effects on normal tissues [59]. In addition, transient or intermittent inhibition may provide another strategy to reduce prolonged disruption of HSP90-dependent proteostasis in normal cells while retaining antitumor activity. However, the present study used genetic HSP90AA1 depletion in tumor cells and did not evaluate systemic pharmacological inhibition, normal-tissue toxicity, or a dose-dependent therapeutic window. Therefore, additional pharmacokinetic, pharmacodynamic, and toxicological studies will be required to determine whether HSP90AA1 can be targeted safely and selectively in vivo.

An additional translational consideration is that genetic depletion of HSP90AA1/Hsp90aa1 and pharmacological HSP90 inhibition should not be regarded as equivalent interventions. In the present study, siRNA-mediated HSP90AA1 knockdown was performed in human breast cancer cells, whereas shRNA-mediated Hsp90aa1 knockdown was used in the mouse 4T1 model to assess the functional contribution of the corresponding gene to the DOX response. In contrast, conventional pharmacological HSP90 inhibitors can interfere with the activity of multiple HSP90 isoforms and destabilize a broad range of HSP90 client proteins, potentially producing biological effects that extend beyond HSP90AA1-specific signaling. Pharmacological HSP90 inhibition may also trigger compensatory stress responses that are not necessarily reproduced by genetic depletion. Therefore, previous studies using HSP90 inhibitors provide biological support for the relevance of the HSP90 chaperone network but should not be interpreted as direct pharmacological validation of the HSP90AA1-associated effects observed in the present study. Translation of these findings will require HSP90AA1-selective inhibitors, degraders, or targeted delivery approaches, together with direct comparison of genetic and pharmacological perturbation and evaluation of target engagement, downstream client-protein responses, antitumor efficacy, and normal-tissue toxicity.

The practical implementation of HSP90AA1 targeting will also depend on the therapeutic modality used. HSP90AA1-directed siRNA or antisense oligonucleotides could provide greater target specificity and more closely reproduce the genetic depletion investigated in the present study, but their application in solid tumors may be limited by delivery efficiency, stability, cellular uptake, and off-target tissue exposure. Conventional small-molecule HSP90 inhibitors are more readily administered but generally affect multiple HSP90 isoforms and client proteins and may therefore provide less HSP90AA1-specific activity. In this context, tumor-targeted nanodelivery may offer a potentially attractive strategy by combining molecular specificity with preferential tumor accumulation and controlled or stimulus-responsive drug release. Recent studies have demonstrated the feasibility of responsive nanoplatforms for targeted therapeutic delivery and tumor microenvironment modulation, including a CXCR4-targeted, pH-responsive nanoplatform developed for triple-negative breast cancer [60]. More broadly, microenvironment-responsive nanotherapeutic systems illustrate how disease-associated local signals can be exploited to achieve spatially restricted therapeutic activation [61]. Thus, tumor-targeted delivery of HSP90AA1-specific siRNA or antisense agents, together with the future development of HSP90AA1-selective inhibitors or degraders, may represent more selective strategies than non-specific systemic HSP90 inhibition. However, these approaches remain to be directly evaluated for HSP90AA1 targeting in breast cancer.

This study has several limitations. First, the bioinformatic analyses were mainly based on retrospective public datasets, and additional validation in independent clinical cohorts is needed. Second, although HSP90AA1 protein expression was validated in 28 paired breast cancer and adjacent non-tumor tissues, the clinical sample size was relatively limited. Detailed information regarding prior anthracycline exposure and treatment response was also not systematically available for all patients in this retrospective cohort, precluding reliable analysis of the association between HSP90AA1 protein expression and clinical DOX response. Therefore, the present study cannot establish HSP90AA1 as a predictive biomarker for DOX benefit, and independent treatment-annotated cohorts, prospective validation, and comparison with established clinicopathological and molecular predictors will be required before any biomarker application can be proposed. Third, ICD was evaluated primarily through representative molecular markers, including CALR, HMGB1, and p-eIF2α. These measurements do not establish bona fide ICD, and additional functional assays, including assessment of cell-surface CALR, extracellular ATP and HMGB1 release, dendritic-cell activation, T-cell responses, and functional immunogenicity, would provide stronger evidence [62,63]. Fourth, metabolic phenotyping was not performed; therefore, whether changes in metabolic adaptation mechanistically contribute to the enhanced DOX response following HSP90AA1 depletion remains unknown. Fifth, the present study did not systematically characterize alternative regulated cell-death modalities, including ferroptosis and pyroptosis, and therefore cannot determine whether these pathways contribute to the effects of HSP90AA1 depletion and DOX treatment. Sixth, immune-related changes in the in vitro experiments were assessed mainly by protein marker expression, which cannot reflect immune-cell infiltration or function. Similarly, immune-associated changes in animal tumors were assessed mainly by Western blotting of CD8A and GZMB, which does not permit quantitative characterization of individual immune-cell populations or definitive identification of their cellular sources. Multiparameter flow cytometry, together with immunohistochemistry or immunofluorescence, will be required to define the abundance, phenotype, spatial distribution, and functional status of tumor-infiltrating immune populations. Seventh, the functional experiments were performed using parental MDA-MB-231 and 4T1 breast cancer models rather than established DOX-resistant derivatives. Therefore, although HSP90AA1/Hsp90aa1 knockdown enhanced the response to DOX in the models examined here, the present data do not establish whether this pathway directly contributes to acquired DOX resistance. Studies using matched parental and DOX-resistant cell lines and syngeneic tumor models will be required to address this question. Moreover, the present study did not systematically compare the effects of HSP90AA1 targeting across different molecular subtypes of breast cancer, and its subtype-specific therapeutic relevance therefore remains to be established. In addition, genetic HSP90AA1/Hsp90aa1 depletion cannot be assumed to reproduce the biological effects of pharmacological HSP90 inhibition, and direct comparison using HSP90AA1-selective pharmacological approaches will be required for therapeutic translation. Finally, the present data support several interconnected associations rather than a definitive causal HSP90AA1–DOX–ICD–immune remodeling cascade. Mechanistic rescue experiments, functional ICD assays, detailed immune-cell characterization in immunocompetent models, and validation in clinically relevant systems will be required to establish the directionality and causality of the proposed model.

In conclusion, this study identifies HSP90AA1 as a potential ICD-related regulator associated with prognosis, doxorubicin response, and immune microenvironment features in breast cancer. HSP90AA1 knockdown enhanced doxorubicin-induced apoptotic responses and ICD-related molecular changes in vitro and strengthened the antitumor effect of doxorubicin in vivo. These effects were accompanied by increased CALR and HMGB1 expression, enhanced CD8A and GZMB expression, and altered PD-L1-associated immune regulatory signaling. Collectively, our findings suggest that targeting HSP90AA1 may represent a potential strategy to improve the response to doxorubicin and promote antitumor immune remodeling in breast cancer.

## 5. Conclusions

In conclusion, this study identifies HSP90AA1 as a candidate molecular factor associated with prognosis, doxorubicin response, and ICD-related features in breast cancer. HSP90AA1 knockdown enhanced doxorubicin-induced apoptotic responses and ICD-related molecular changes in vitro and strengthened the antitumor response to doxorubicin in vivo. These effects were accompanied by increased CALR and HMGB1 expression, increased CD8A and GZMB levels, and altered PD-L1-associated immune regulatory signaling. Collectively, our findings suggest that HSP90AA1 may modulate cellular adaptation to doxorubicin and that its depletion is associated with enhanced apoptotic, ICD-related, and immune-associated molecular responses. Further functional studies are required to establish whether HSP90AA1 directly regulates bona fide ICD and to define the contribution of metabolic adaptation and other regulated cell-death pathways to this response.

## Data Availability

The public datasets analyzed in this study are available from The Cancer Genome Atlas Breast Invasive Carcinoma cohort (TCGA-BRCA). The remaining data generated or analyzed during the current study, including Western blotting and experimental validation data, are available from the corresponding author upon reasonable request.

## Supplementary Materials

The following supporting information can be downloaded at: https://www.mdpi.com/article/10.3390/cancers18182913/s1, Figure S1: Expression patterns of ICD subtype-related differentially expressed genes. (A) Heatmap showing the expression profiles of representative differentially expressed genes between ICD-low and ICD-high breast cancer subtypes; Figure S2: Correlation analysis of tumor-infiltrating immune cell populations. (A) Correlation matrix showing the relationships among 22 estimated tumor-infiltrating immune cell types in breast cancer samples. Red indicates positive correlation, whereas blue indicates negative correlation. Figure S3: Validation of stable Hsp90aa1 knockdown incultured 4T1cells. (A) Western blot analysis of HSP90AA1 protein expression in cultured 4T1-shNC and 4T1-shHsp90aa1 cells. (B) Quantification of HSP90AA1 protein expression normalized to GAPDH. Data are presented as mean ± SD. ** *p* < 0.01; Table S1: Antibodies used for Western blotting; File S1: WB raw data.
