# Supplementary material for: HSP90AA1 Knockdown Enhances Doxorubicin Sensitivity by Promoting Immunogenic Cell Death-Related Signaling and Immune-Related Tumor Microenvironment Remodeling in Breast Cancer
Source: Cancers (Basel). 2026 Sep 9;18(18):2913. doi: 10.3390/cancers18182913 (PMC13604569; doi:10.3390/cancers18182913)
Supplement: Supplementary file 1 [file cancers-18-02913-s001.zip › Figure S1.pdf]

A

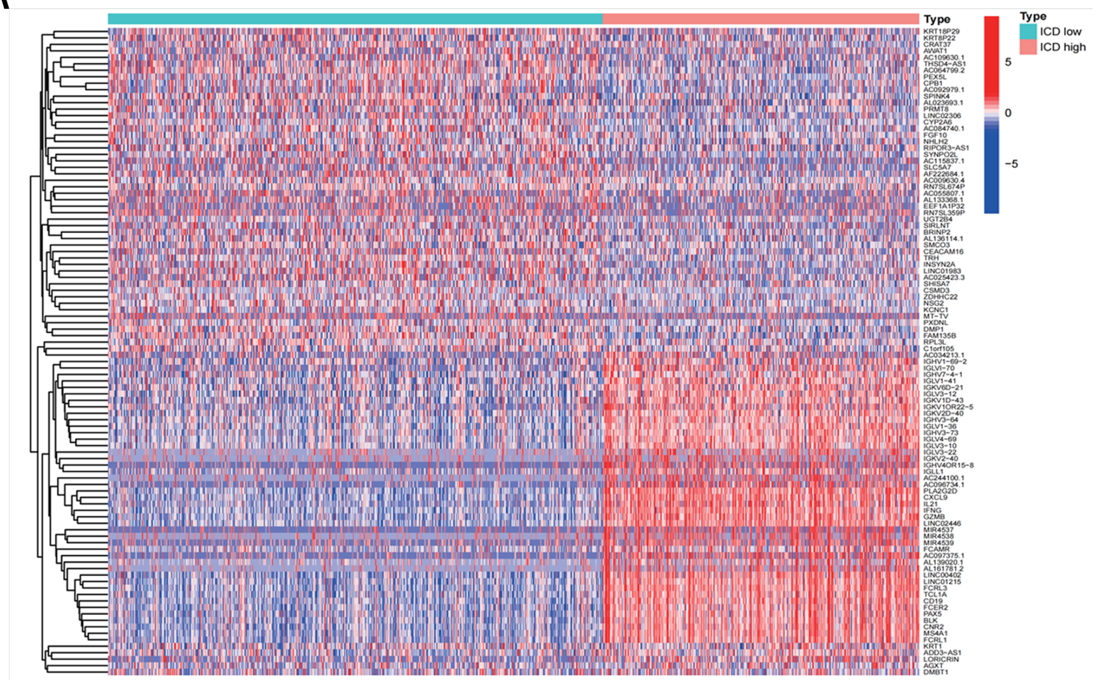

Figure S1. Expression patterns of ICD subtype-related differentially expressed genes.

(A) Heatmap showing the expression profiles of representative differentially expressed genes between ICD-low and ICD-high breast cancer subtypes.
