# Supplementary material for: HSP90AA1 Knockdown Enhances Doxorubicin Sensitivity by Promoting Immunogenic Cell Death-Related Signaling and Immune-Related Tumor Microenvironment Remodeling in Breast Cancer
Source: Cancers (Basel). 2026 Sep 9;18(18):2913. doi: 10.3390/cancers18182913 (PMC13604569; doi:10.3390/cancers18182913)
Supplement: Supplementary file 1 [file cancers-18-02913-s001.zip › Figure S2.pdf]

A

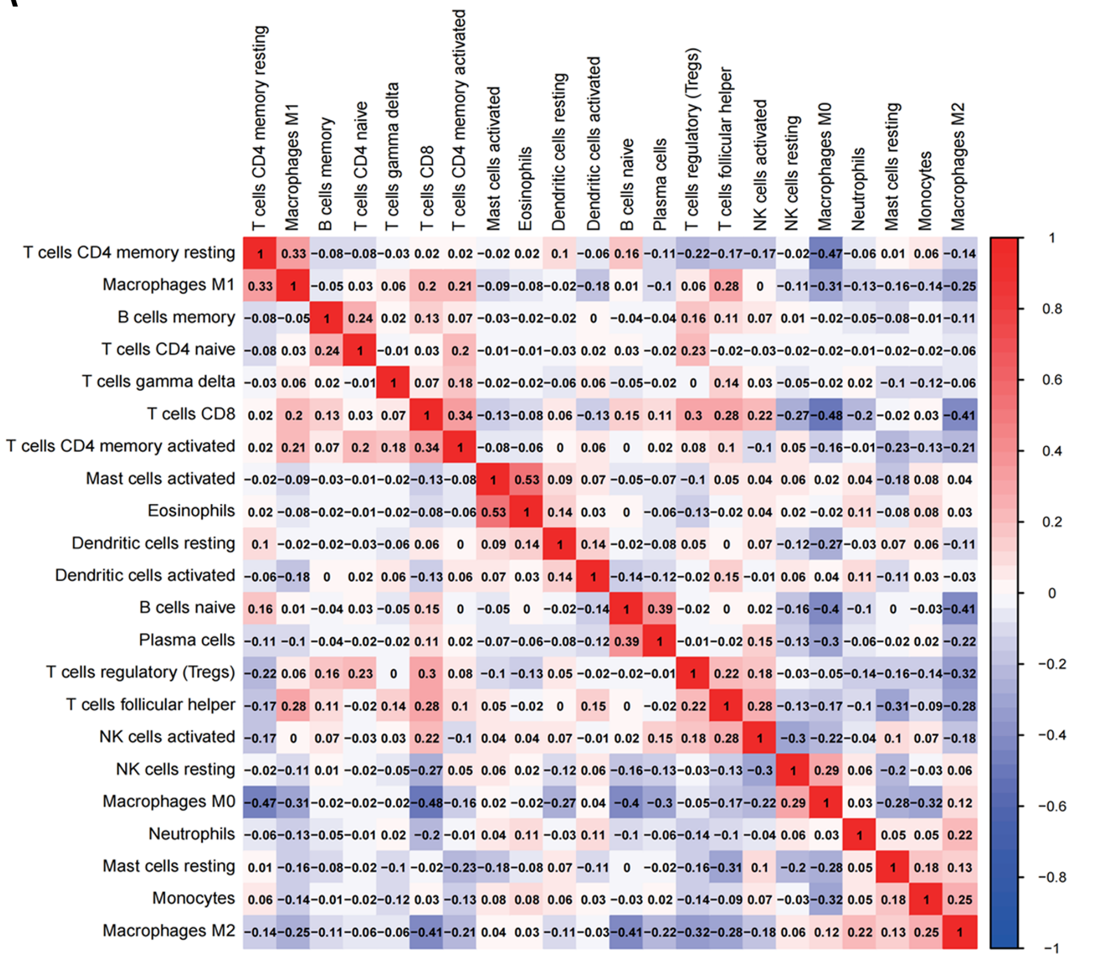

Figure S2. Correlation analysis of tumor-infiltrating immune cell populations.
(A)Correlation matrix showing the relationships among 22 estimated tumor-infiltrating immune cell types in breast cancer samples. Red indicates positive correlation, whereas blue indicates negative correlation.
