# Supplementary material for: HSP90AA1 Knockdown Enhances Doxorubicin Sensitivity by Promoting Immunogenic Cell Death-Related Signaling and Immune-Related Tumor Microenvironment Remodeling in Breast Cancer
Source: Cancers (Basel). 2026 Sep 9;18(18):2913. doi: 10.3390/cancers18182913 (PMC13604569; doi:10.3390/cancers18182913)
Supplement: Supplementary file 1 [file cancers-18-02913-s001.zip › Figure S3.pdf]

A

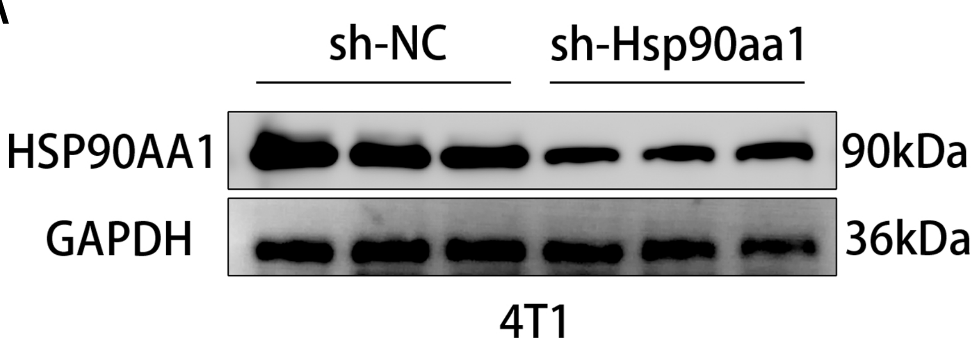


B

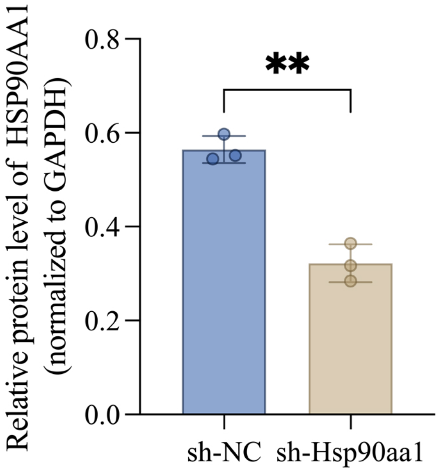


Figure S3. Validation of stable Hsp90aa1 knockdown in cultured 4T1 cells. (A) Western blot analysis of HSP90AA1 protein expression in cultured 4T1-shNC and 4T1-shHsp90aa1 cells. (B) Quantification of HSP90AA1 protein expression normalized to GAPDH. Data are presented as mean ± SD.**P < 0.01.
