# Supplementary material for: HSP90AA1 Knockdown Enhances Doxorubicin Sensitivity by Promoting Immunogenic Cell Death-Related Signaling and Immune-Related Tumor Microenvironment Remodeling in Breast Cancer
Source: Cancers (Basel). 2026 Sep 9;18(18):2913. doi: 10.3390/cancers18182913 (PMC13604569; doi:10.3390/cancers18182913)

The original data is arranged based on the sequence of Western Blots data provided in the manuscript.

Marker: Epizyme，WJ103

Figure 4 J (N1T1-N7T7)

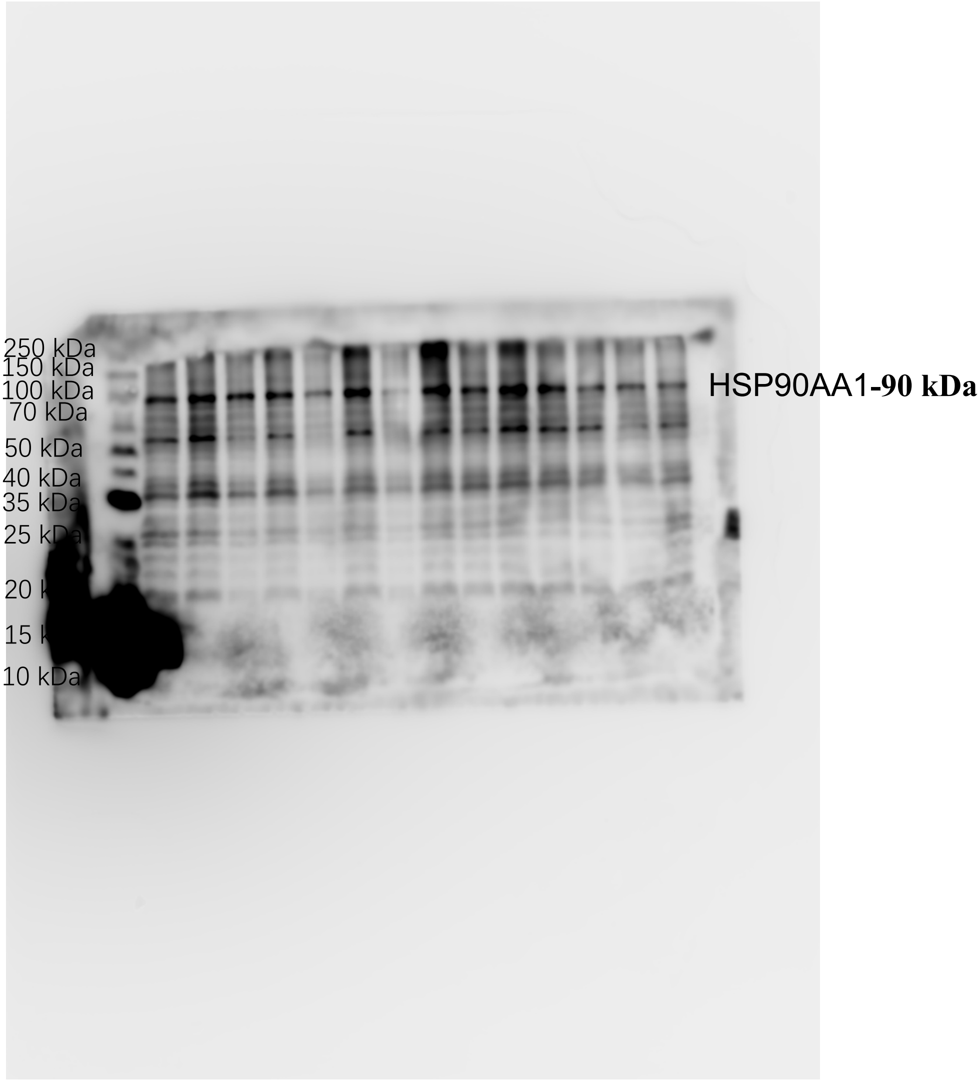

Figure 4J（N8T8-N14T14）

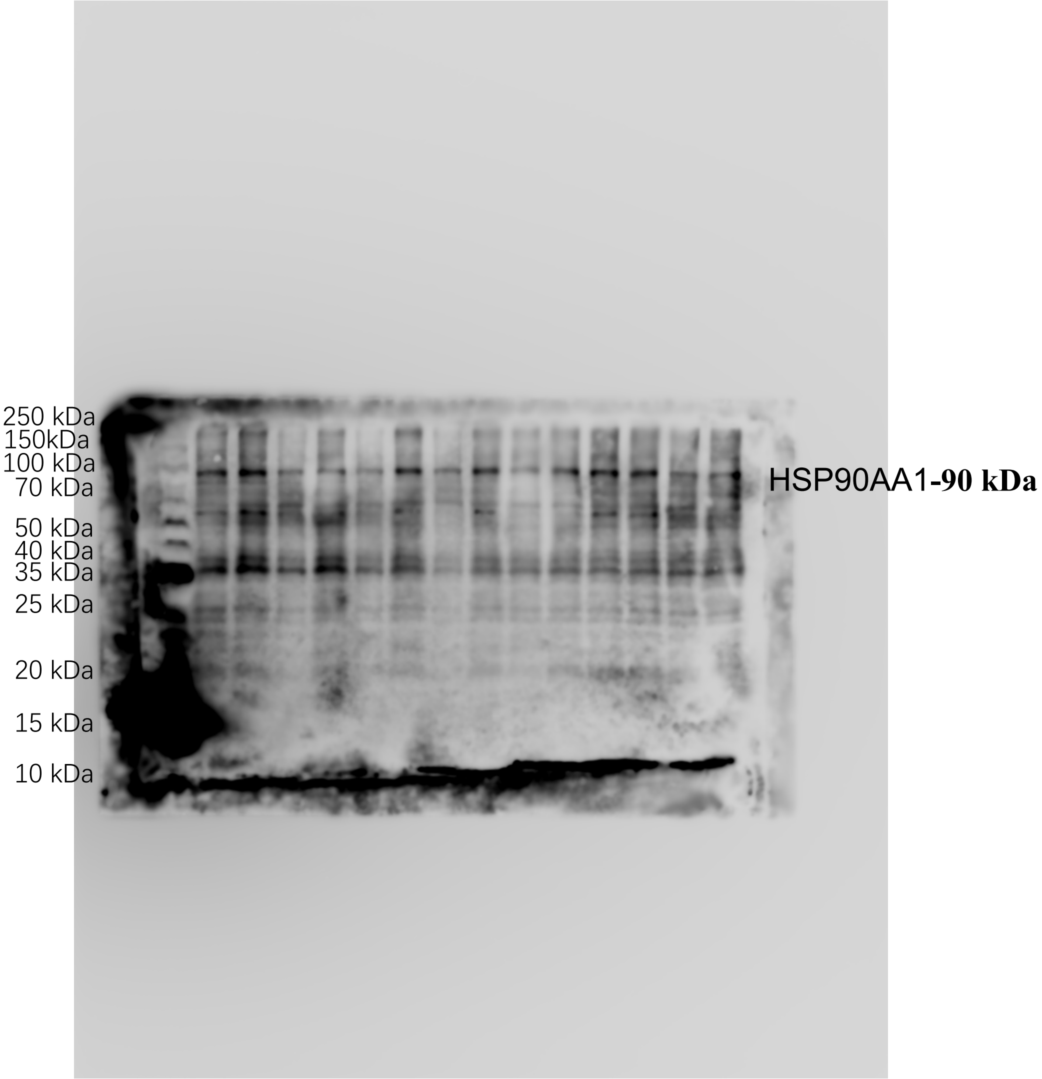

Figure 4 J (N15T15-N21T21)

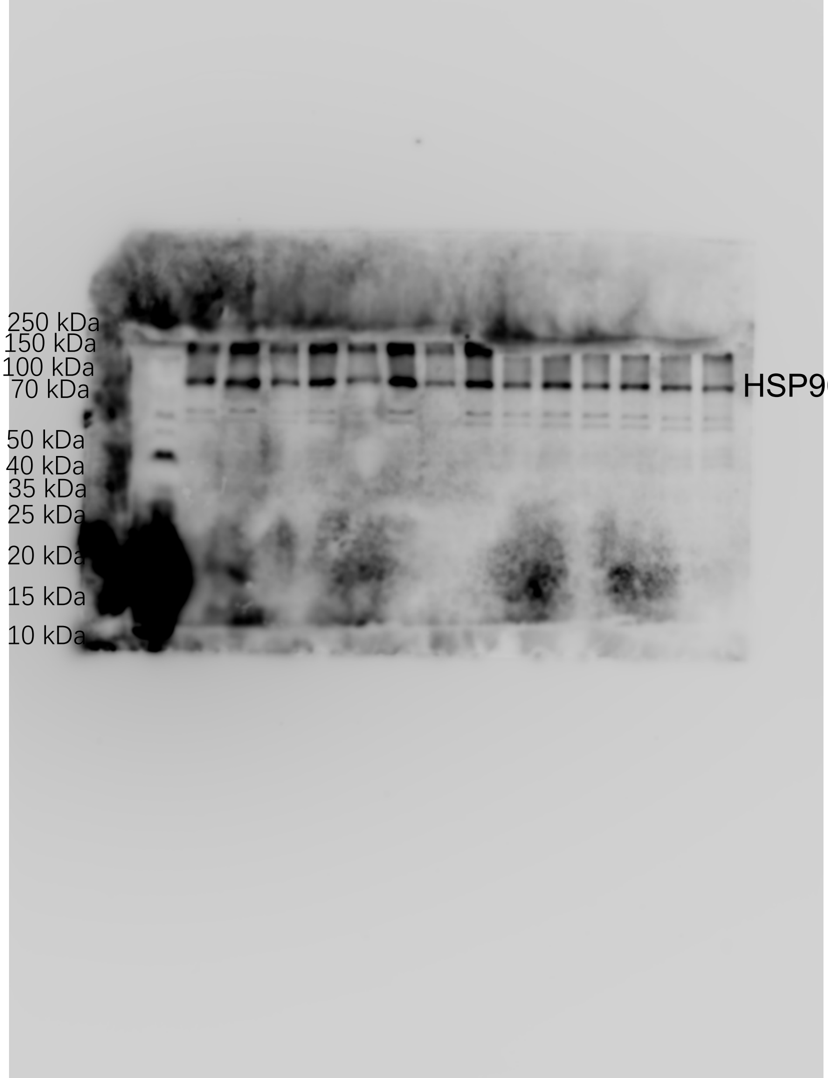

Figure 4 J（N22T22-N28T28）

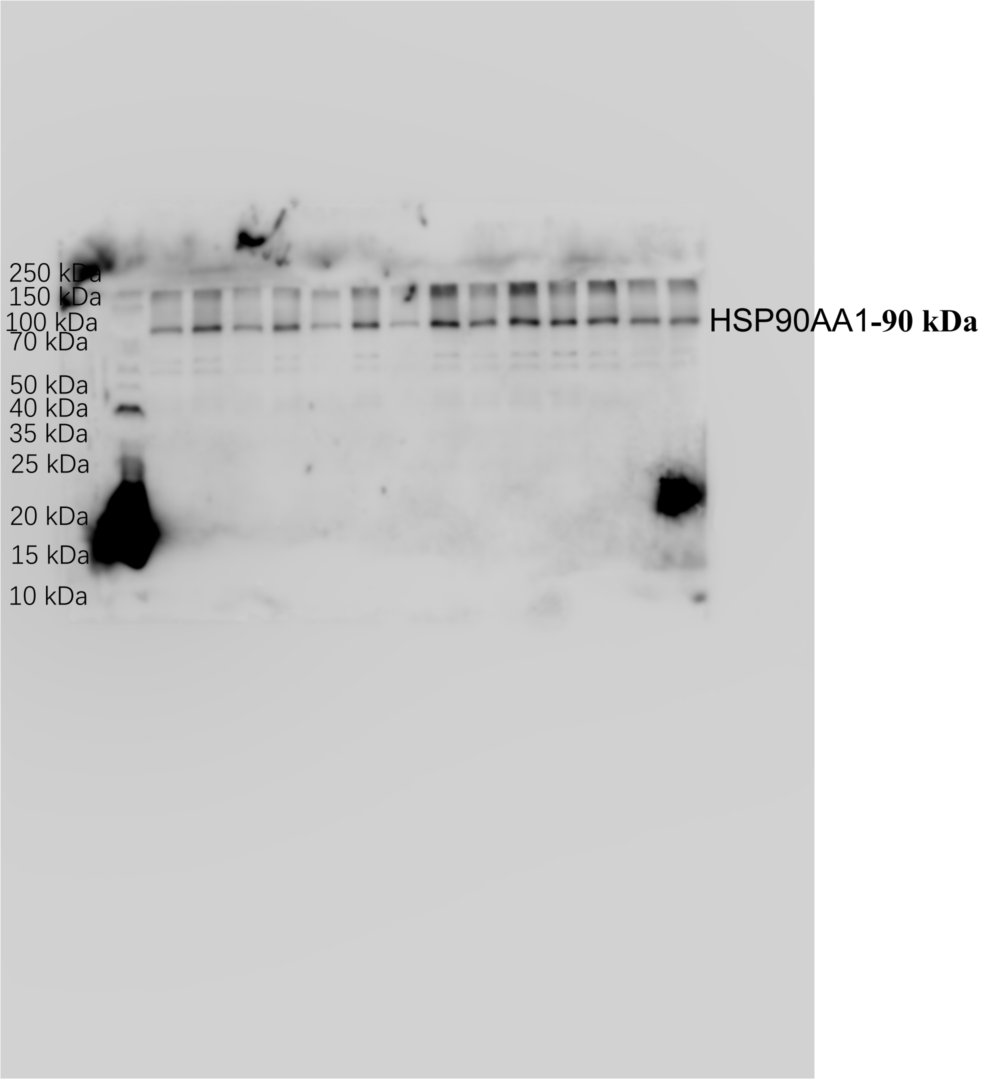

Figure 4 J (N1T1-N7T7)

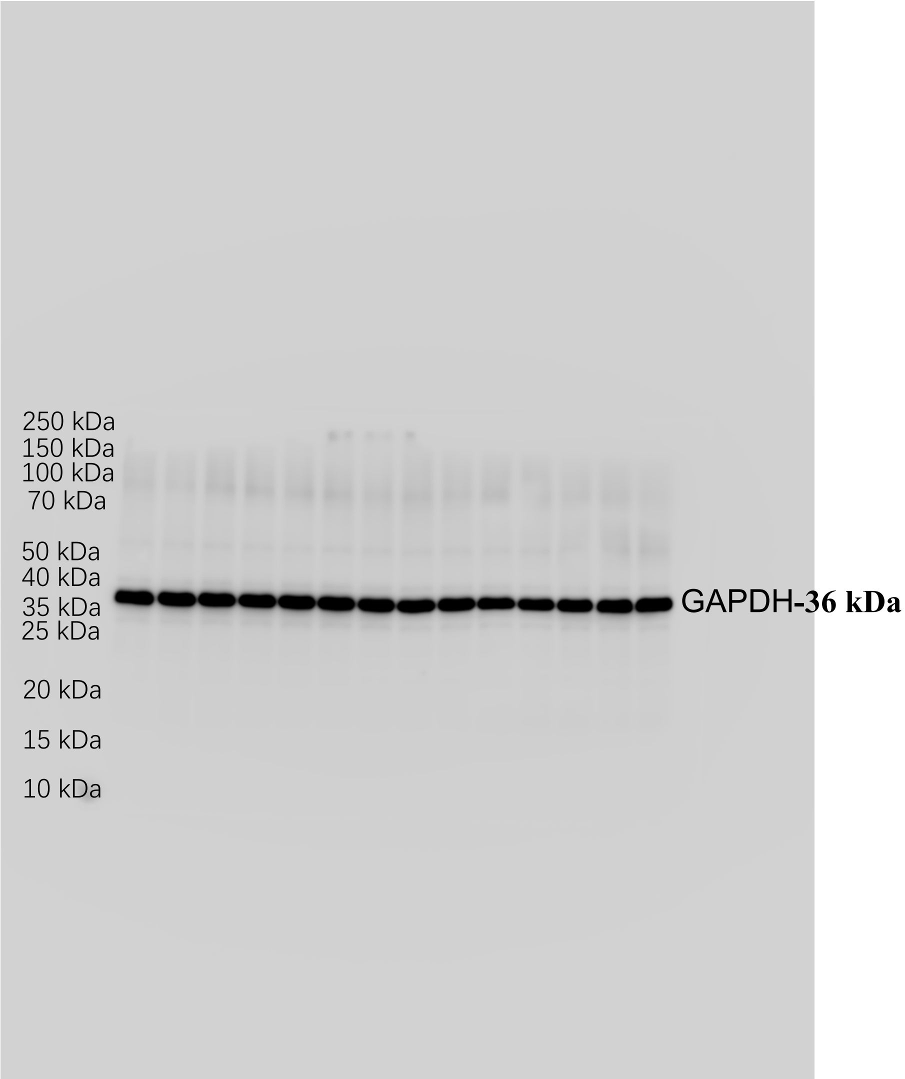

Figure 4J（N8T8-N14T14）

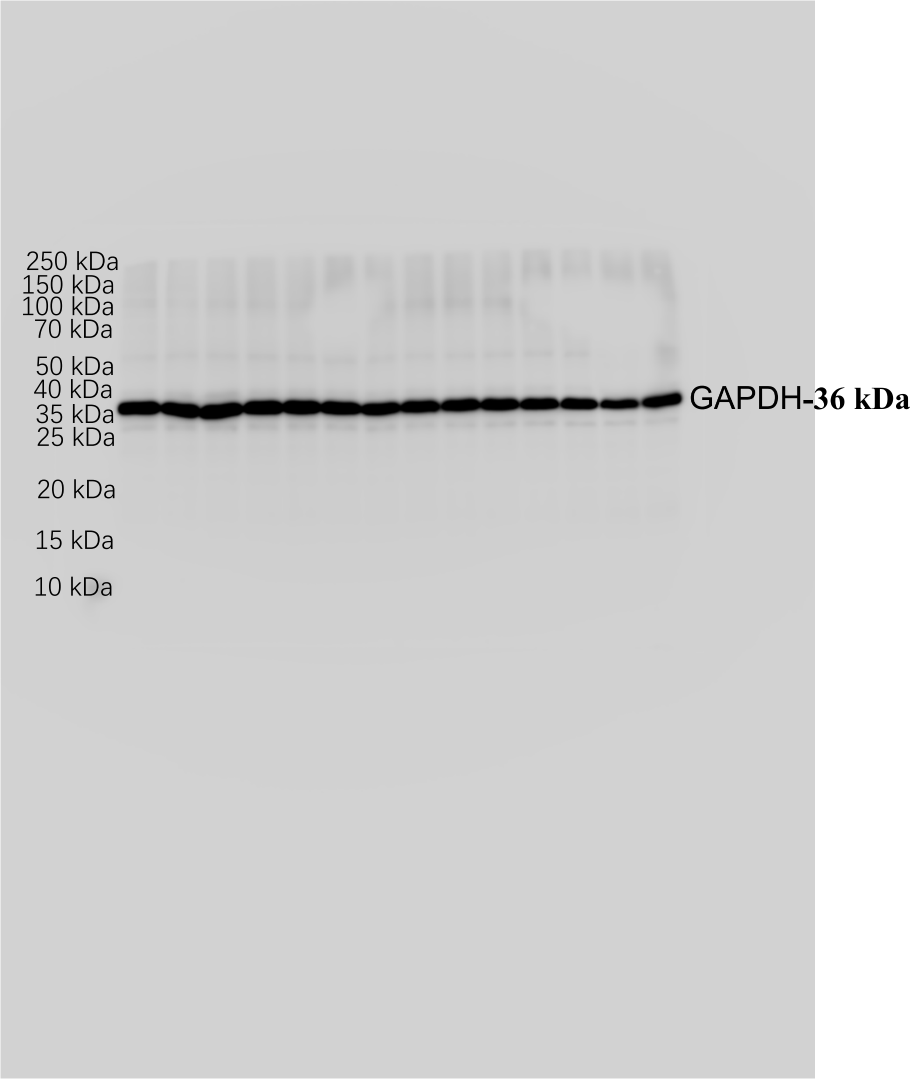

Figure 4J（N15T15-N21T21）

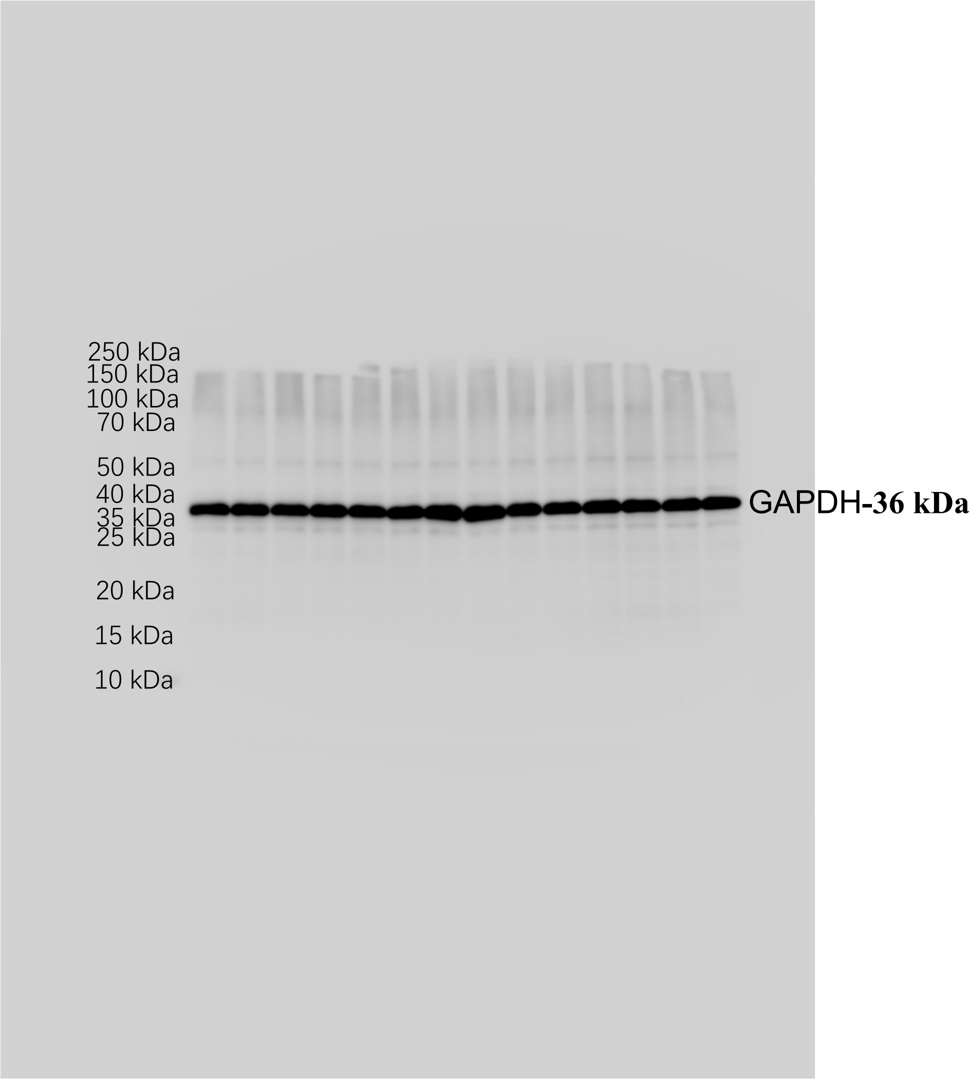

Figure 4J（N22T22-N28T28）

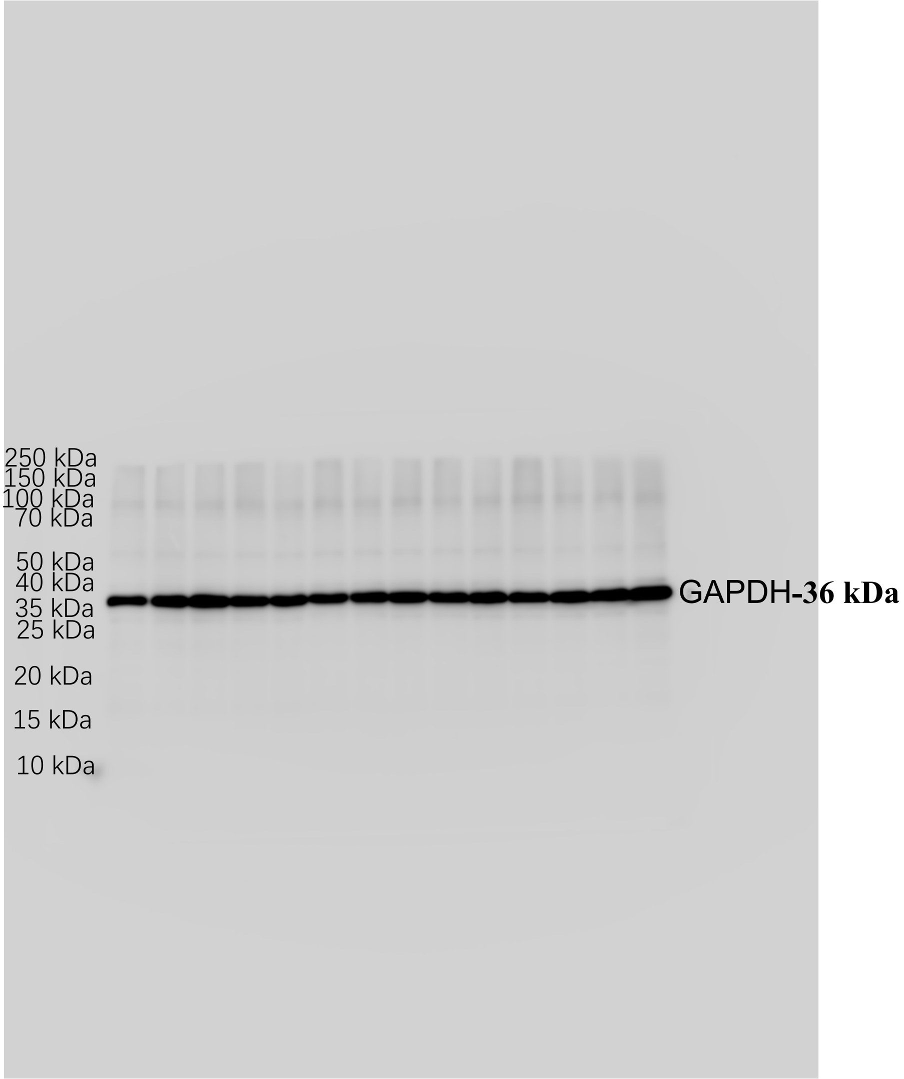

Figure 5 A (HSP90AA1**-90kDa)**

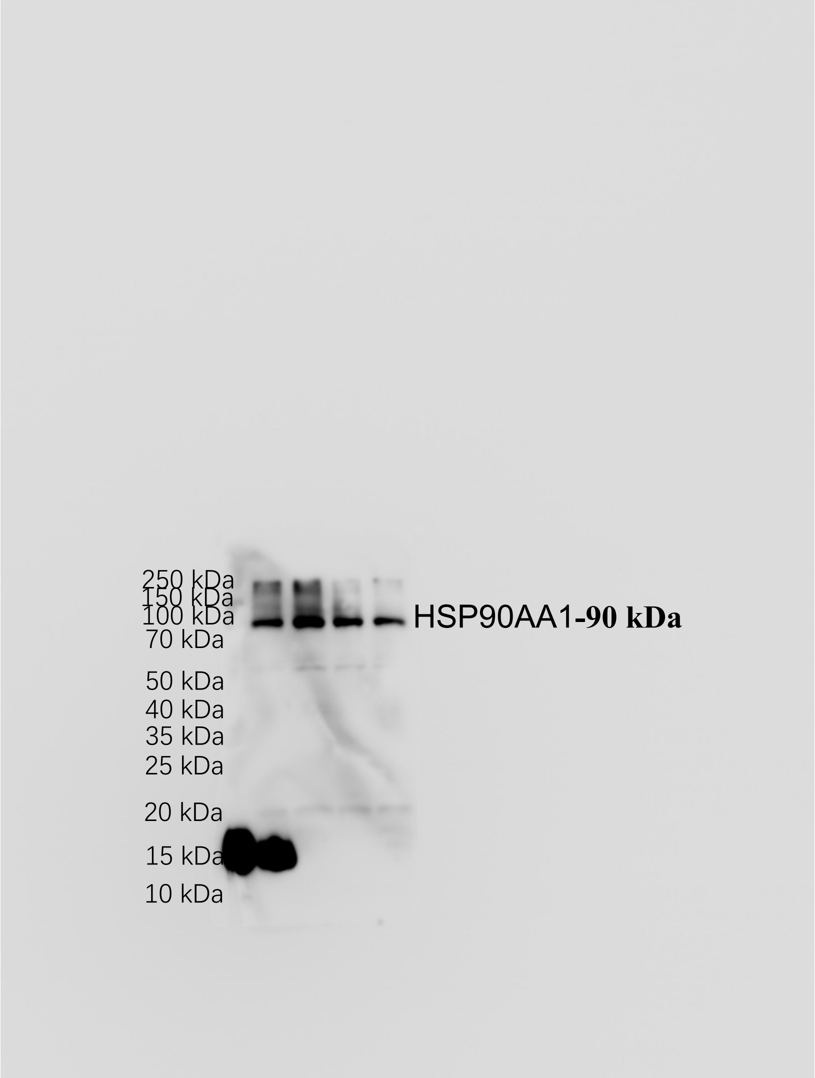

Figure 5 A (GAPDH-36kDa)

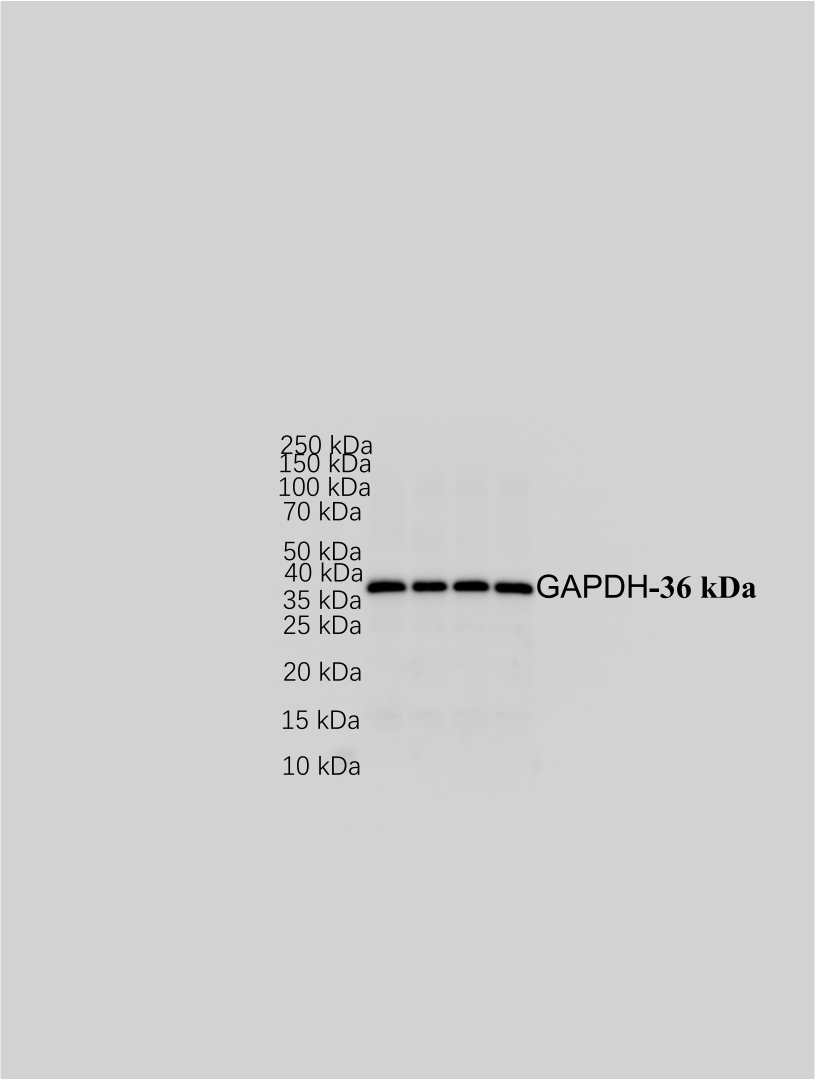

Figure 5D (HSP90AA1-90kDa)

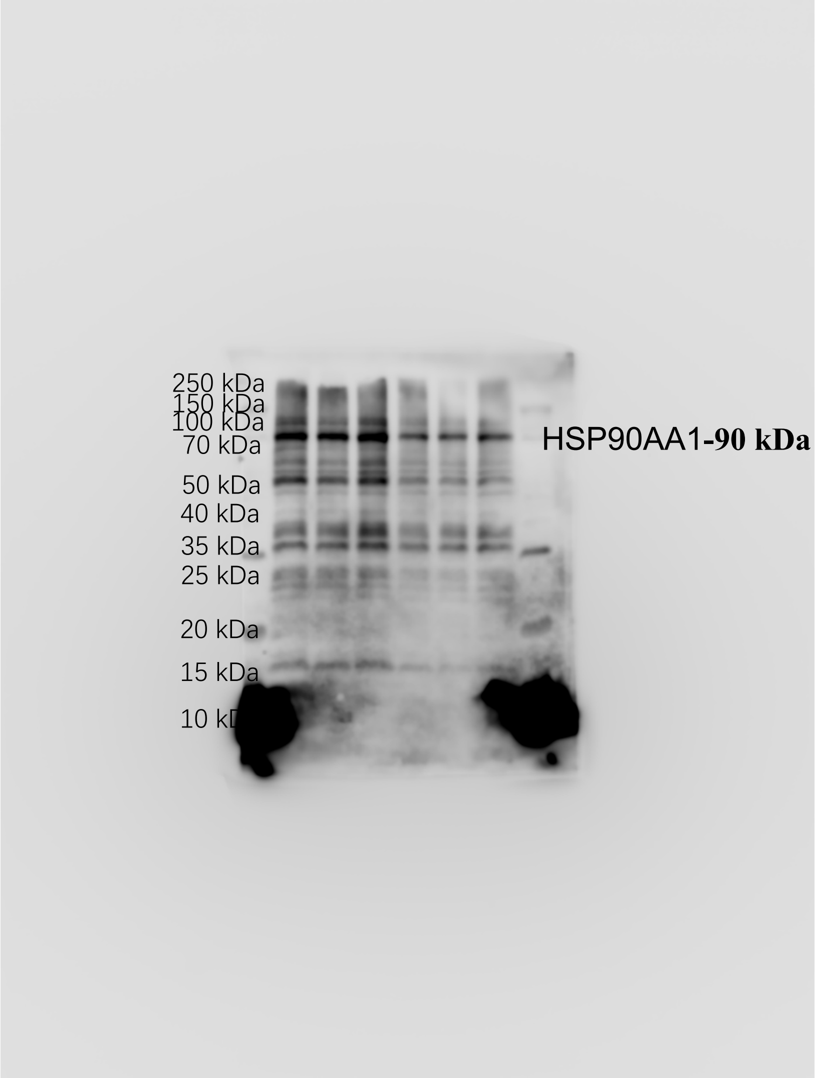

Figure 5D (GAPDH-36kDa)

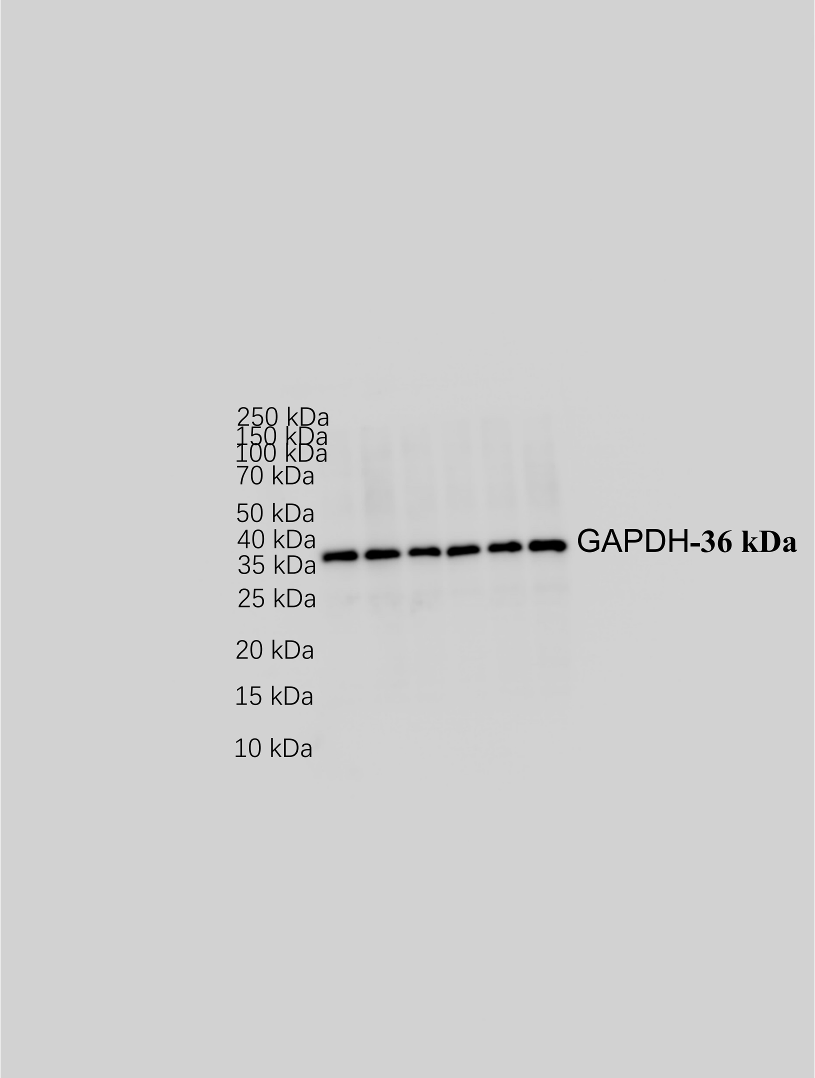

Figure 5F (HSP90AA1**-90kDa)**

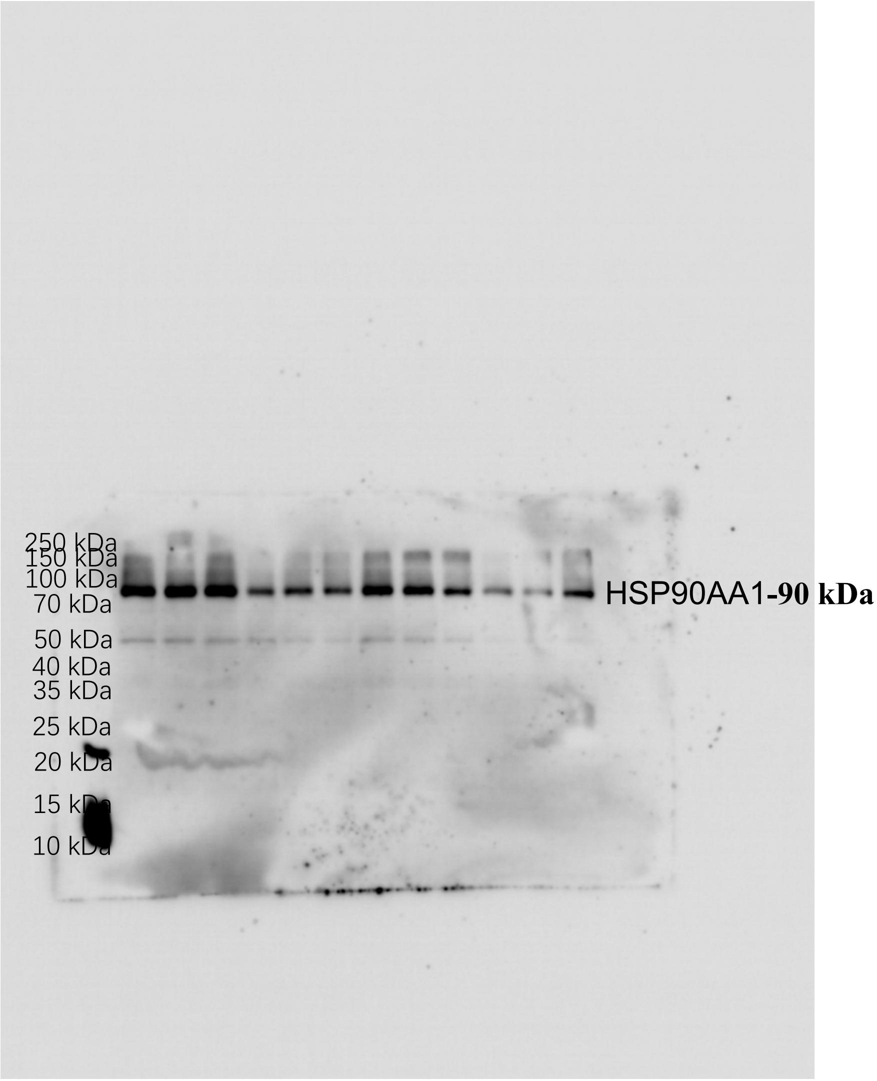

Figure 5F (GAPDH-36kDa)

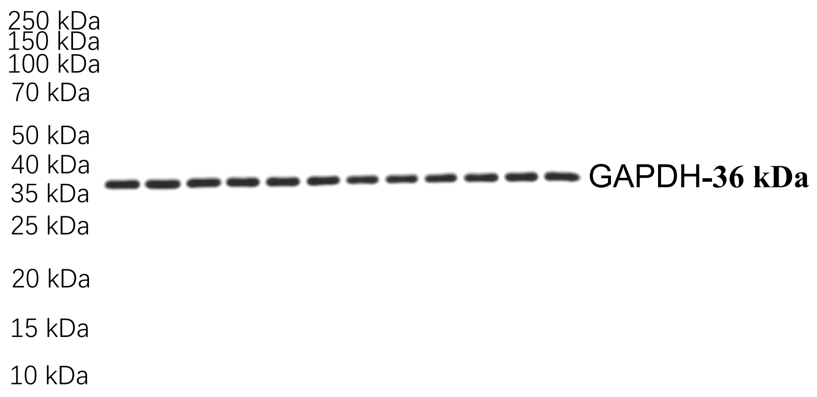

Figure 5F (Bax-21kDa)

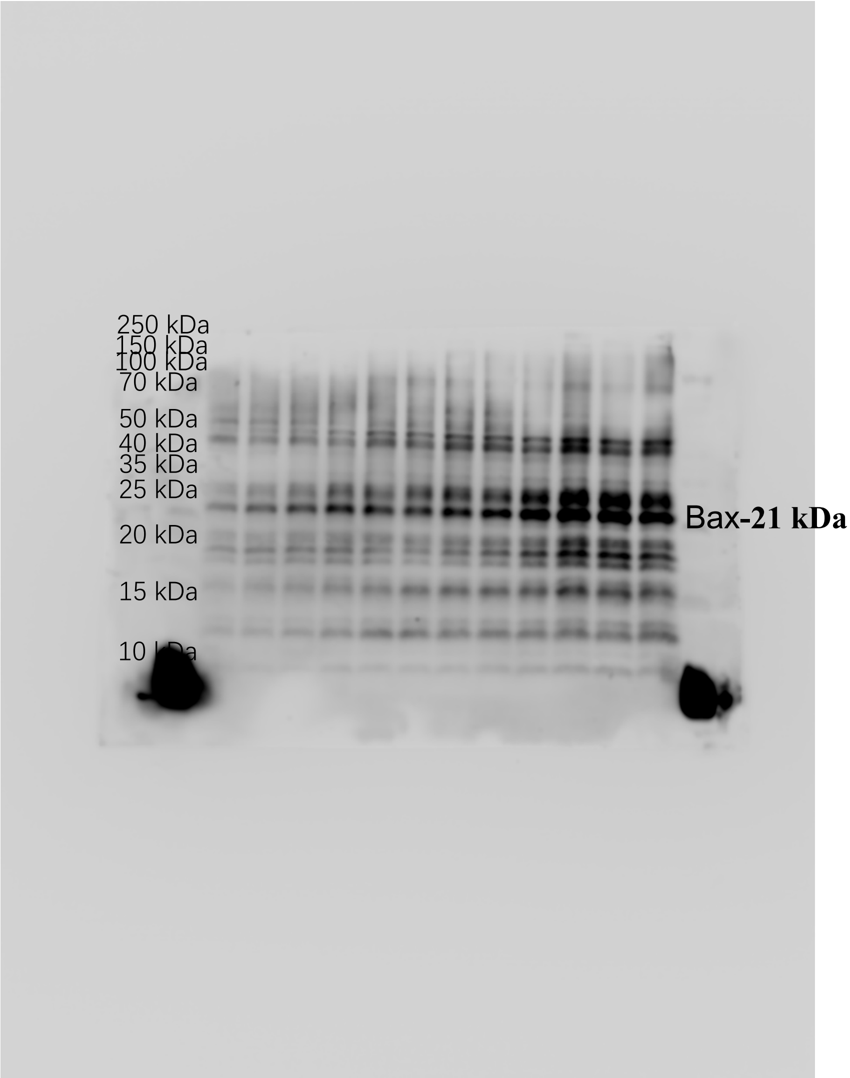

Figure 5F (Bcl-2-26kDa)

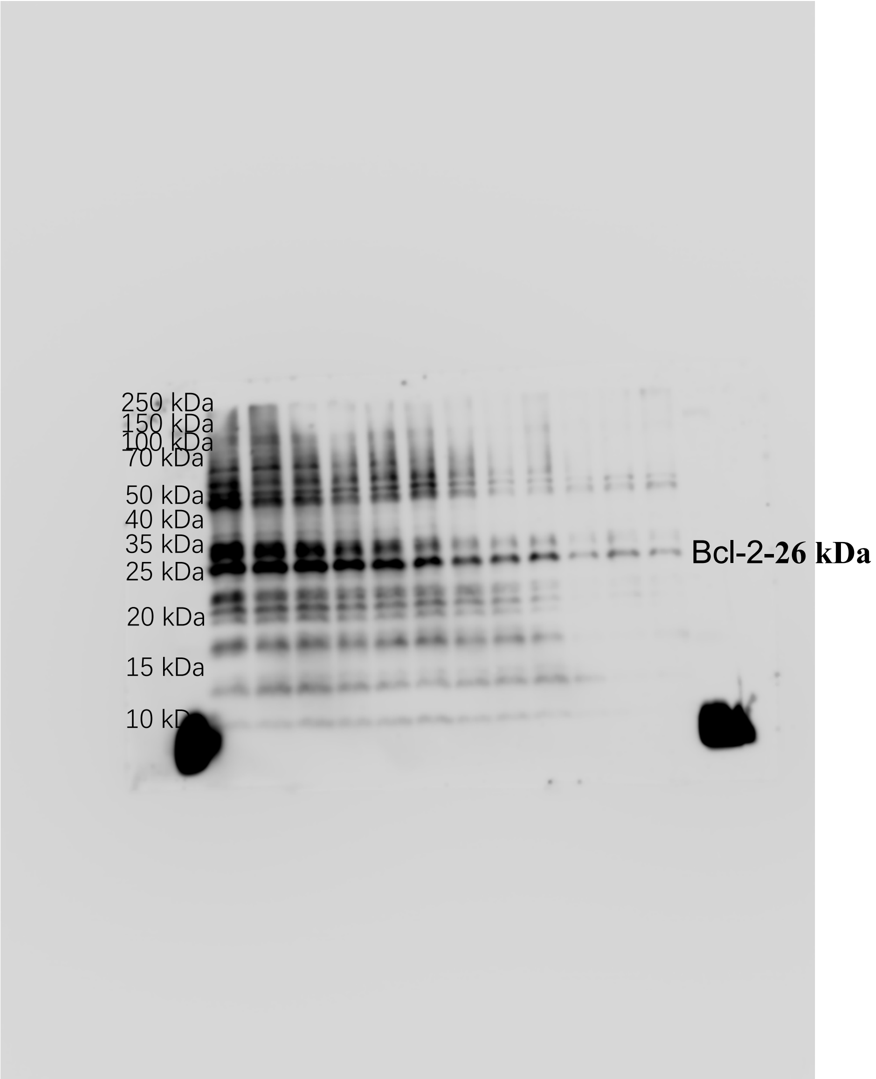

Figure 5F (Cleaved-Caspase-3-19/17-(35)**kDa)**

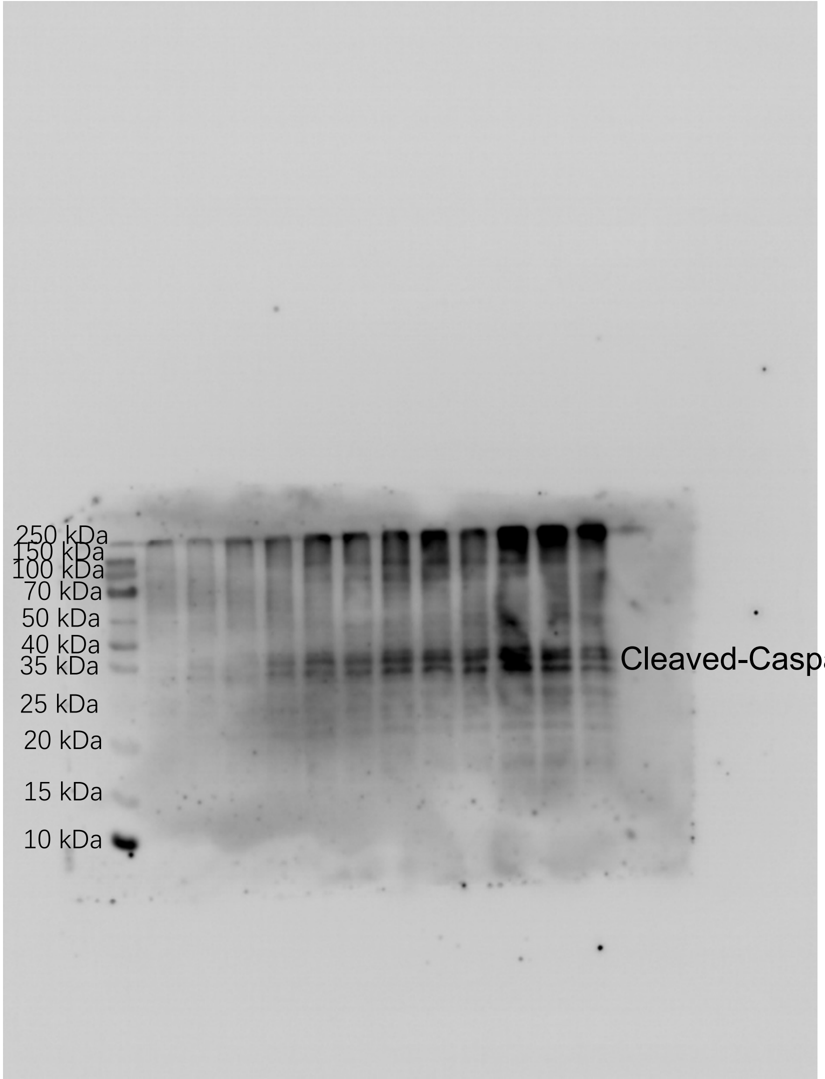

Cleaved-Caspase-3-19/17-(35)**kDa**

Figure5F(GAPDH-36kDa)

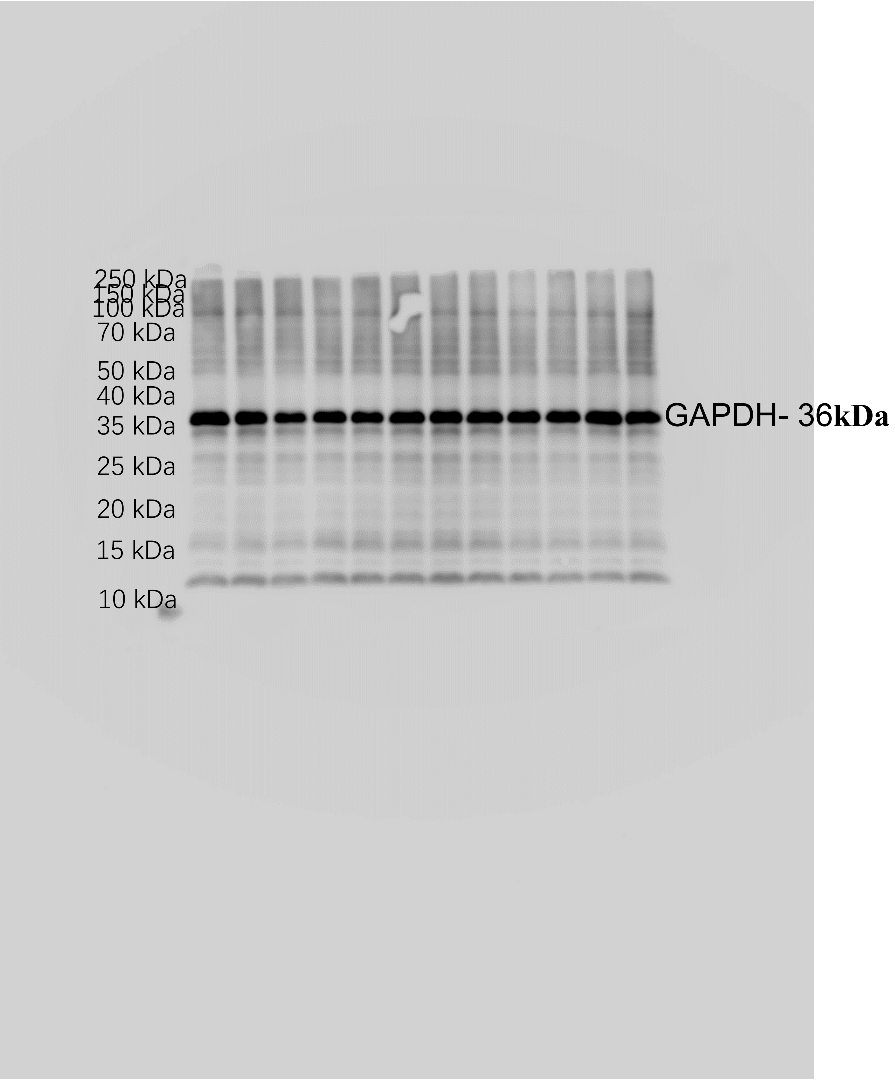

Figure5F(CALR-55kDa)

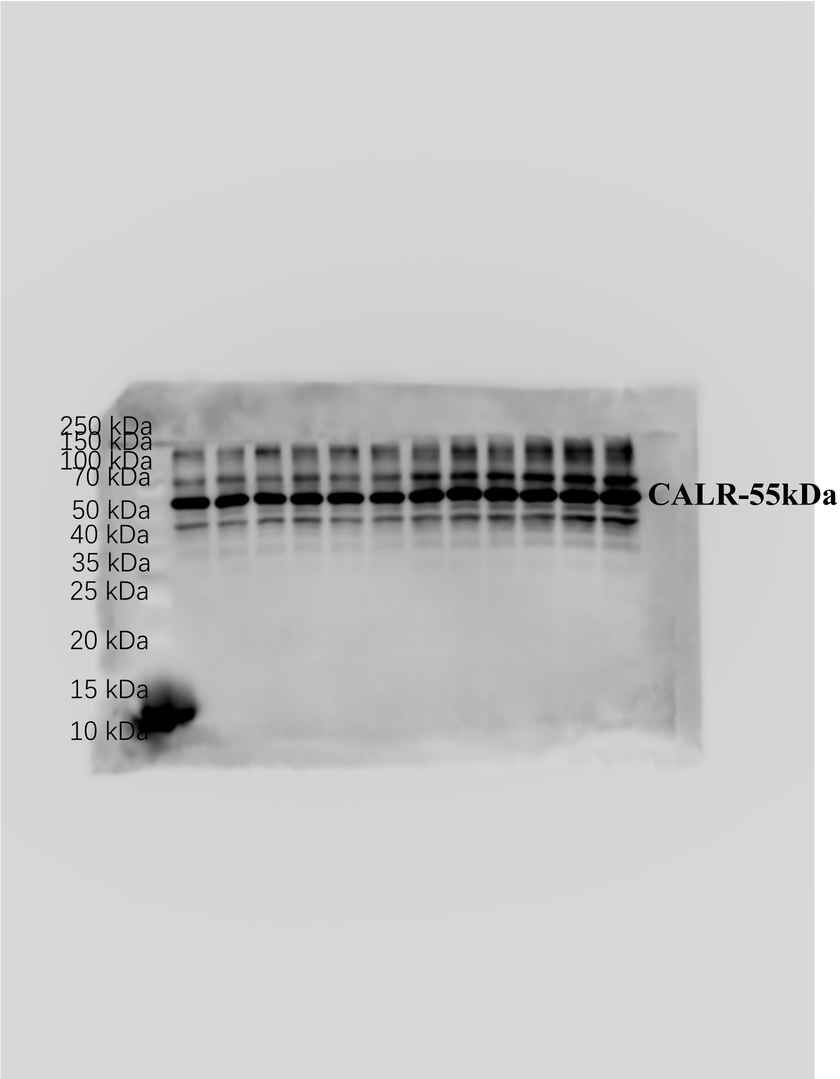

Figure5F(HMGB1-25kDa)

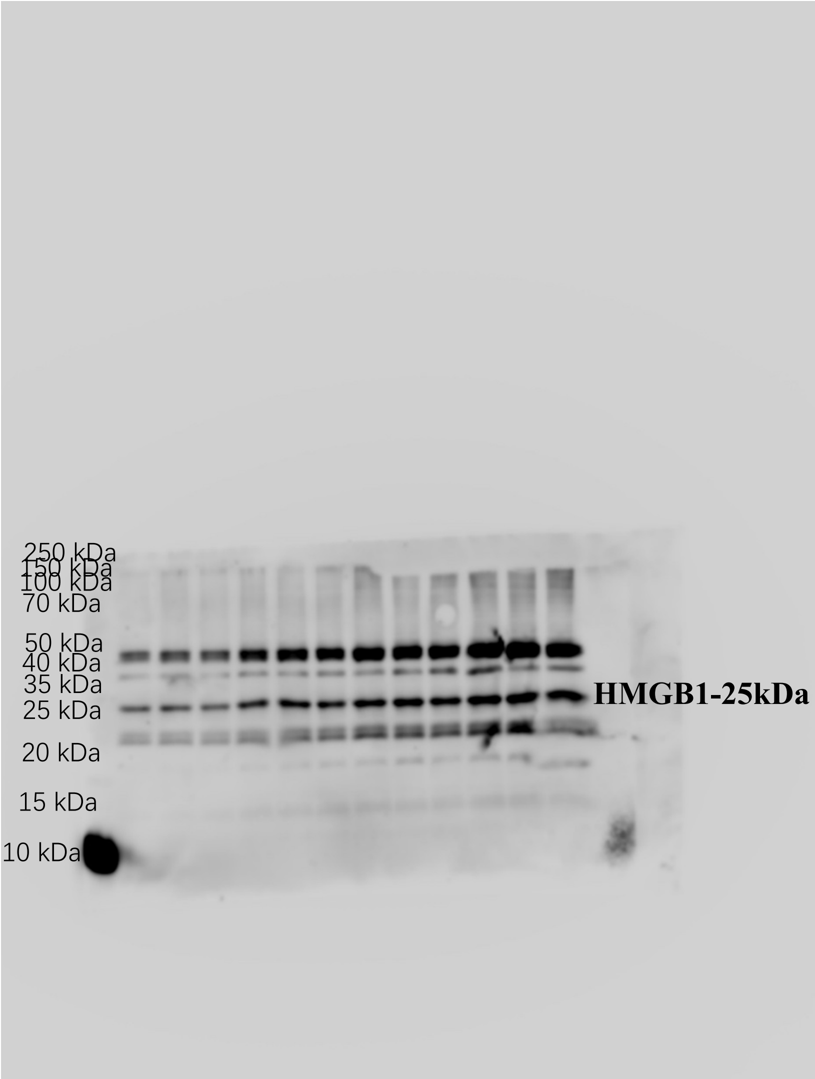

Figure5F(eIF2a-38kDa)

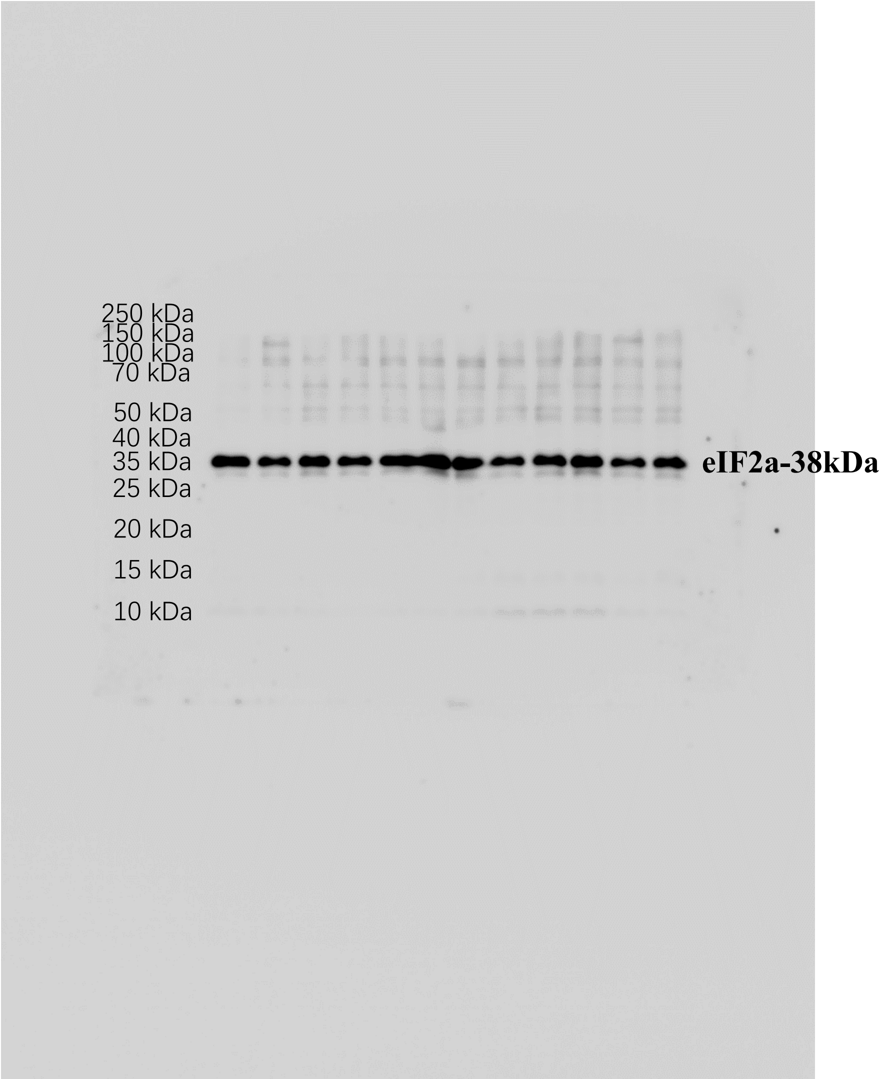

Figure5F(p-eIF2a-38kDa)

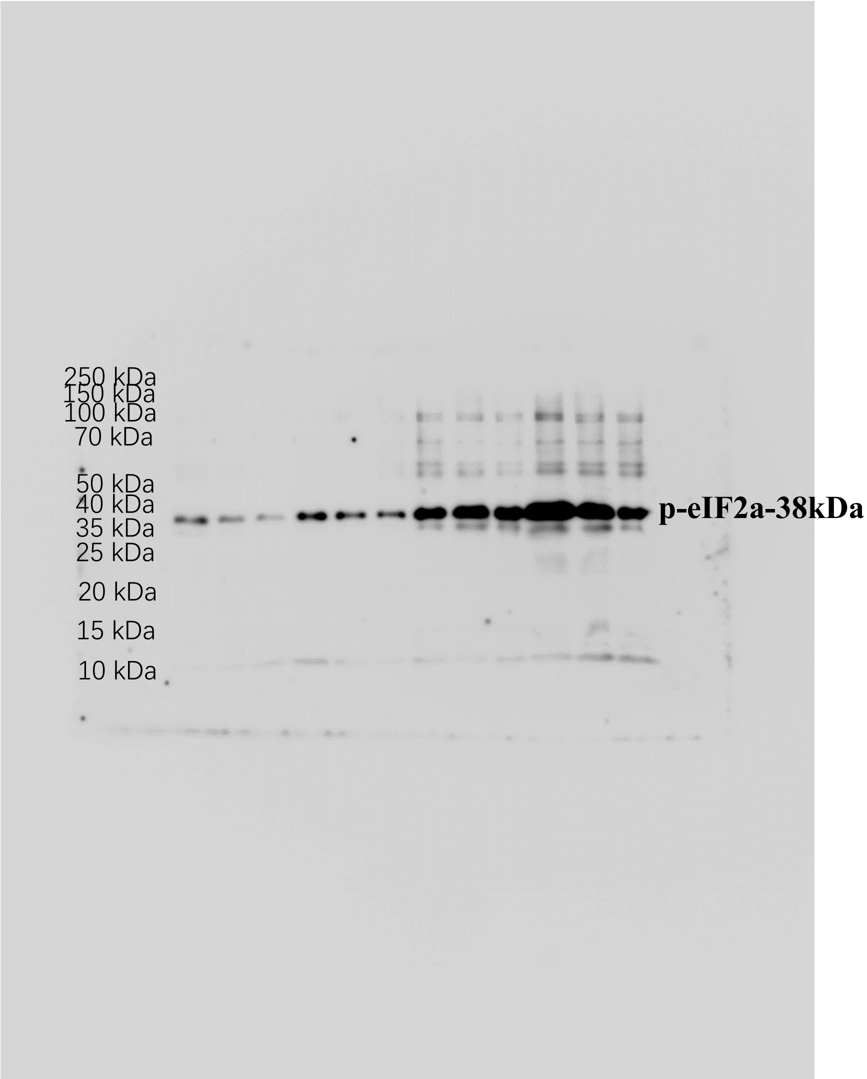

Figure5F(GAPDH-36kDa)

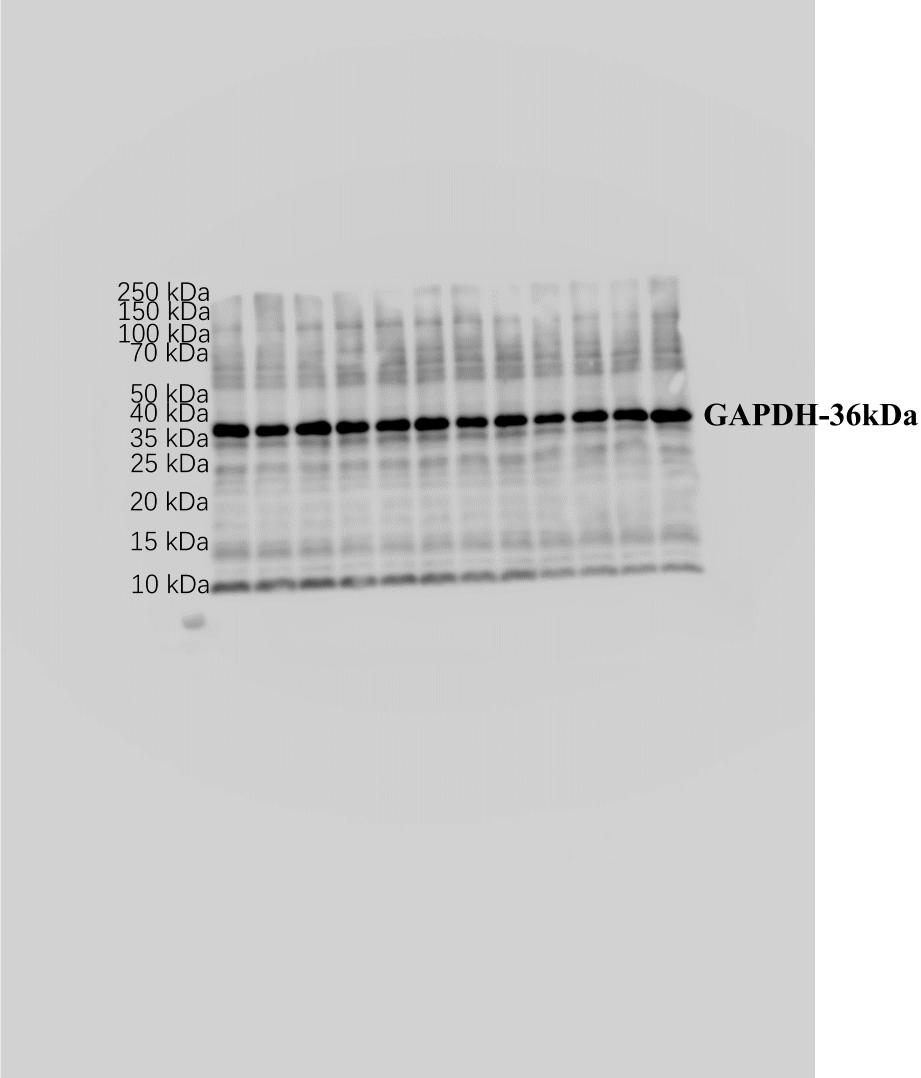

Figure5F(CD8A-(25-32)kDa)

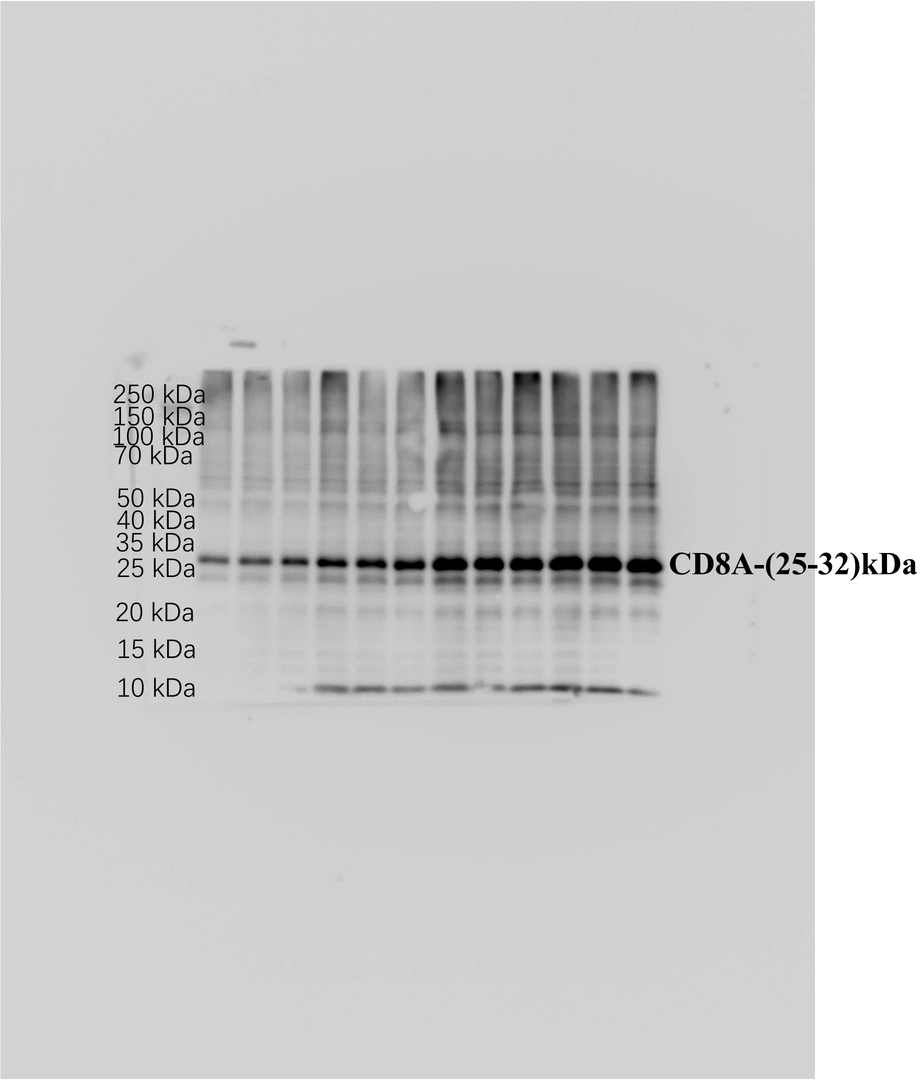

Figure5F(GZMB-(28-32)kDa)

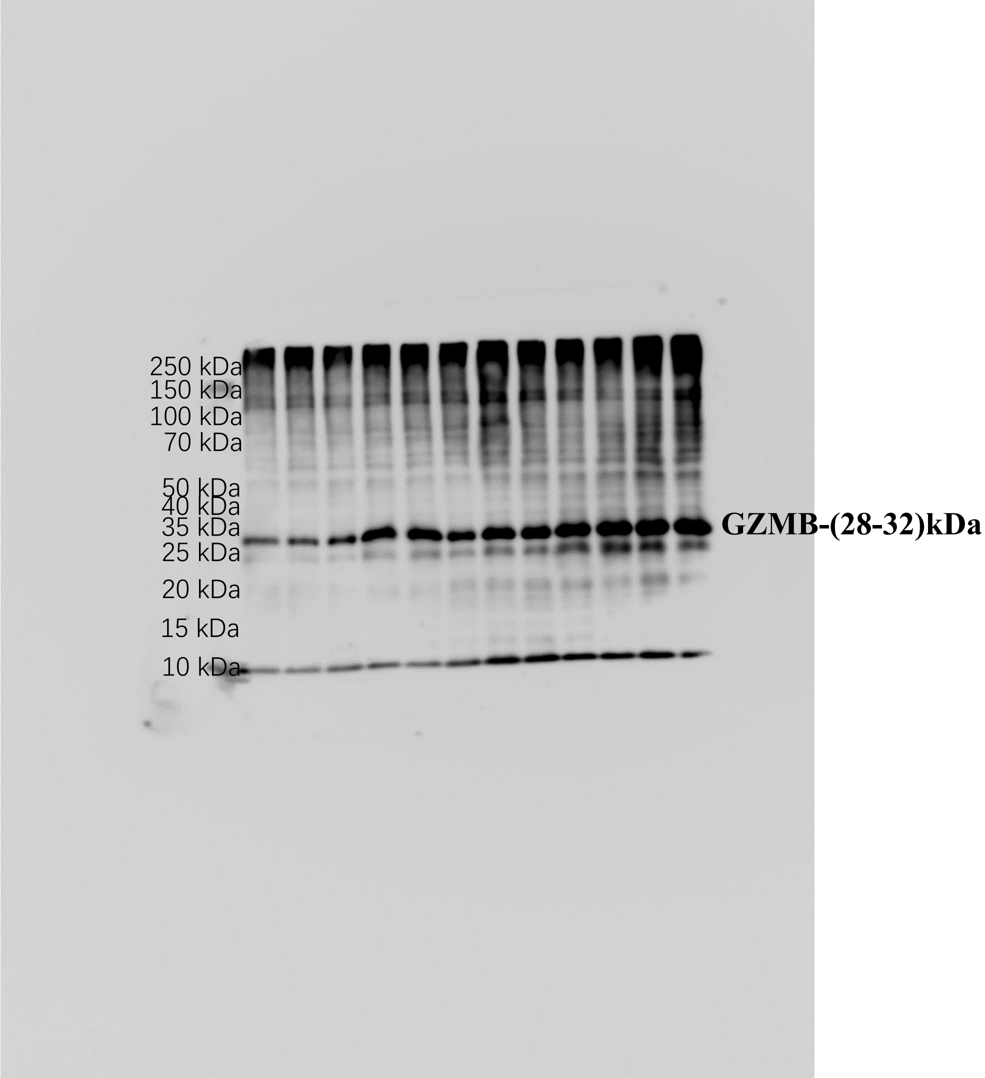

Figure5F(GAPDH-36kDa)

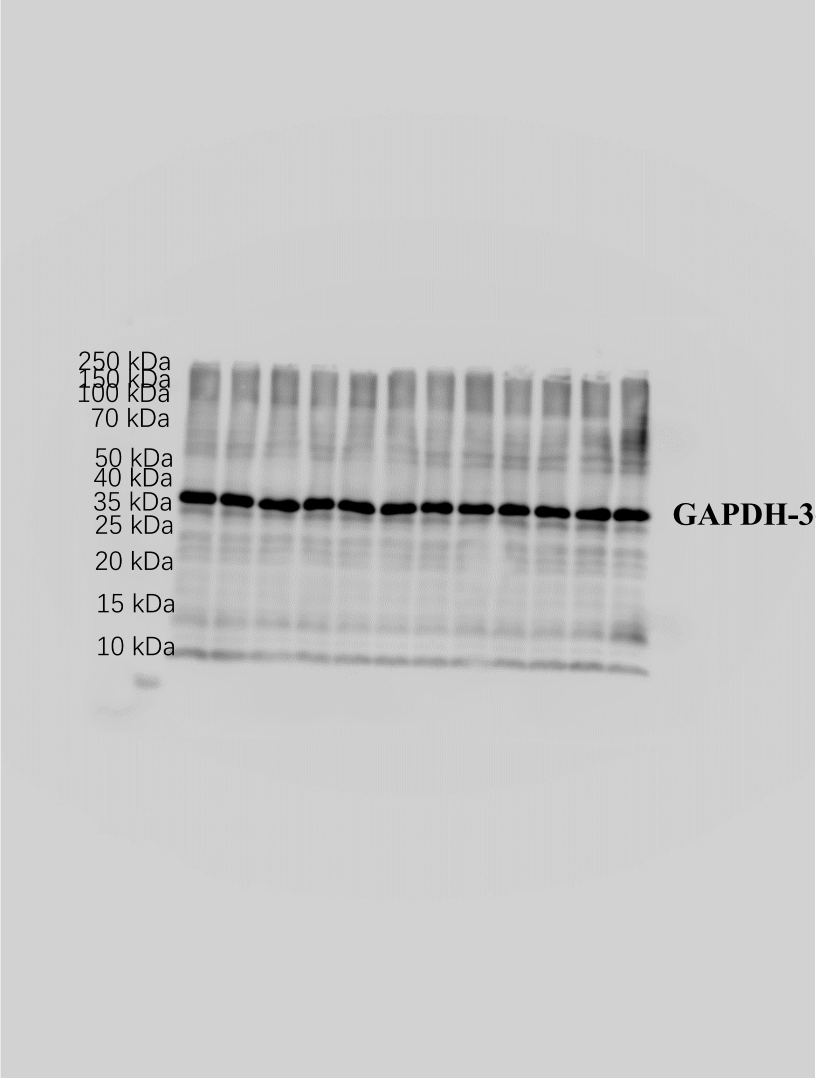

GAPDH-36kDa

Figure5F(FOXP3-(47-50)kDa)

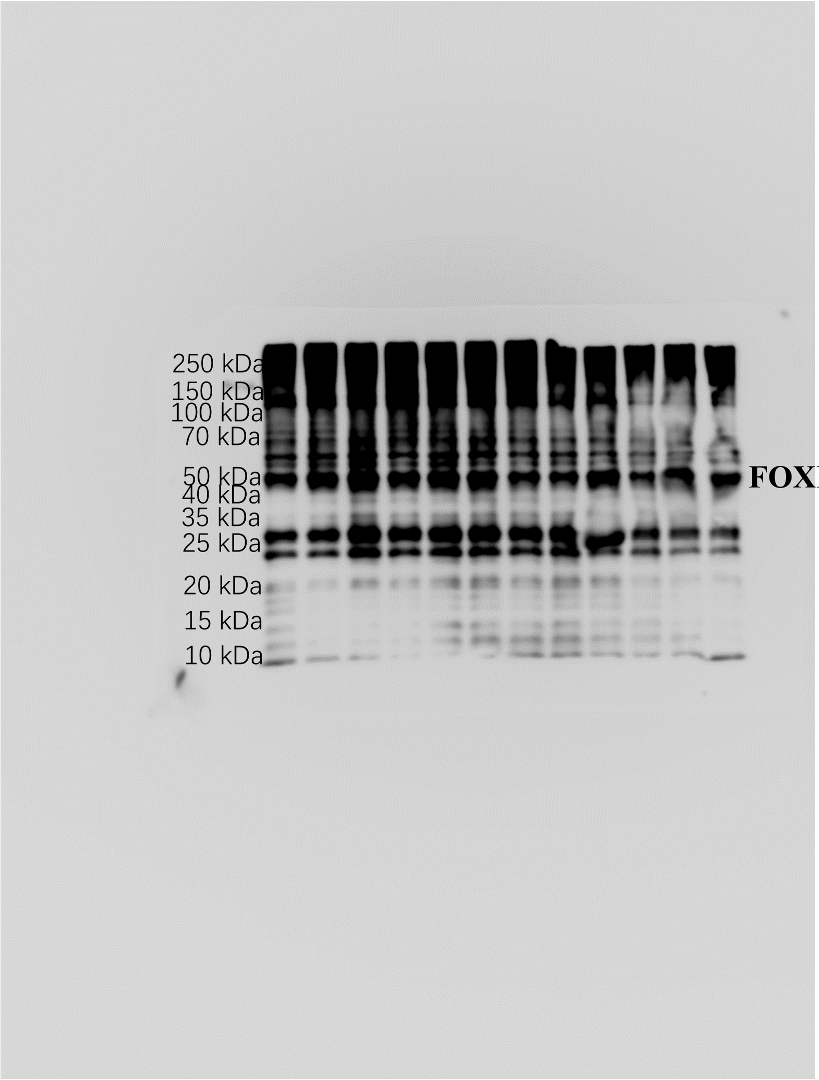

FOXP3-(47-50)kDa

Figure5F(PD-L1-(45-55)kDa)

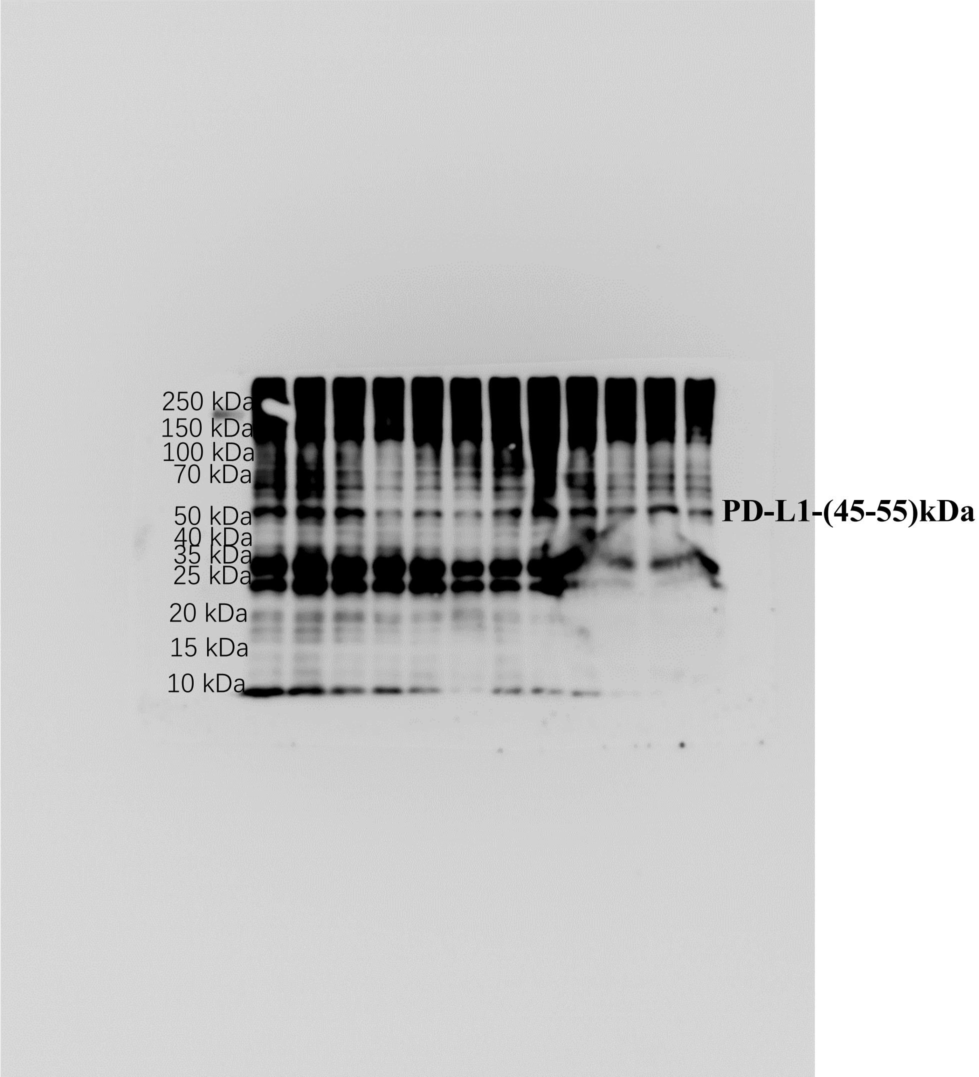

Figure5F(GAPDH-36kDa)

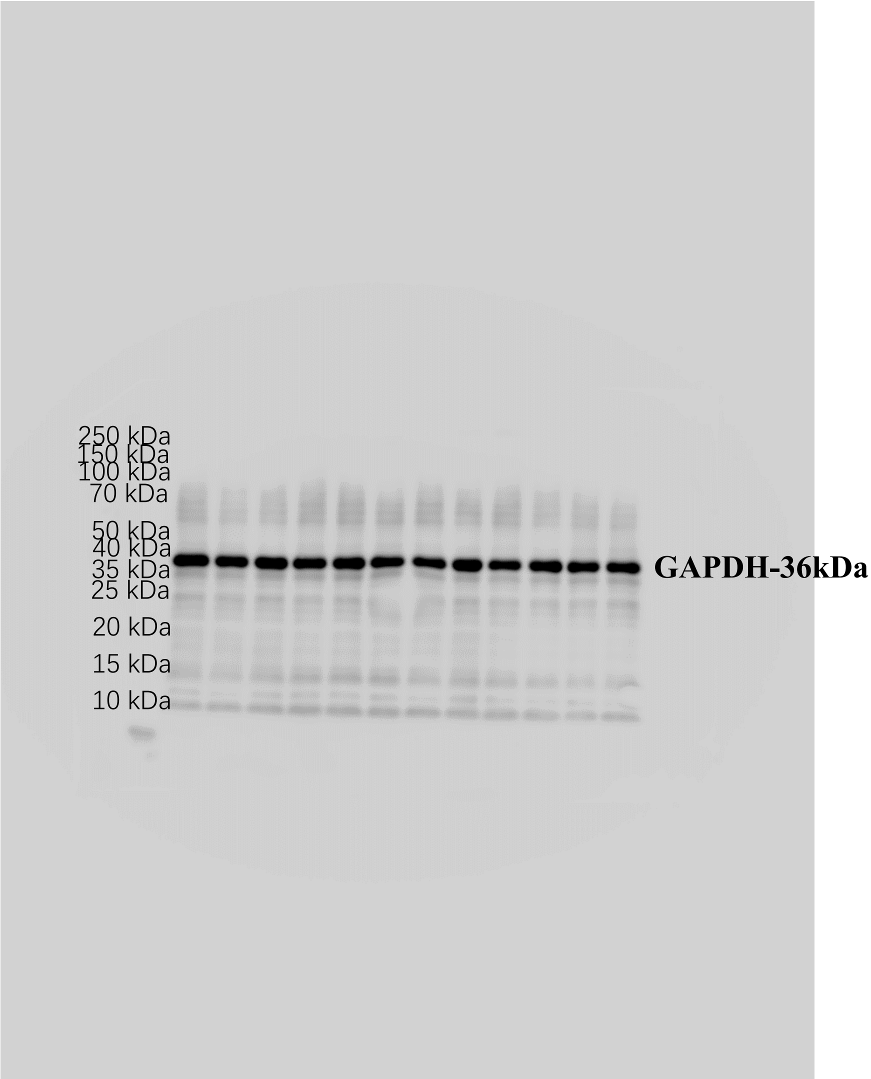

Figure 6D(HSP90AA1-90kDa)

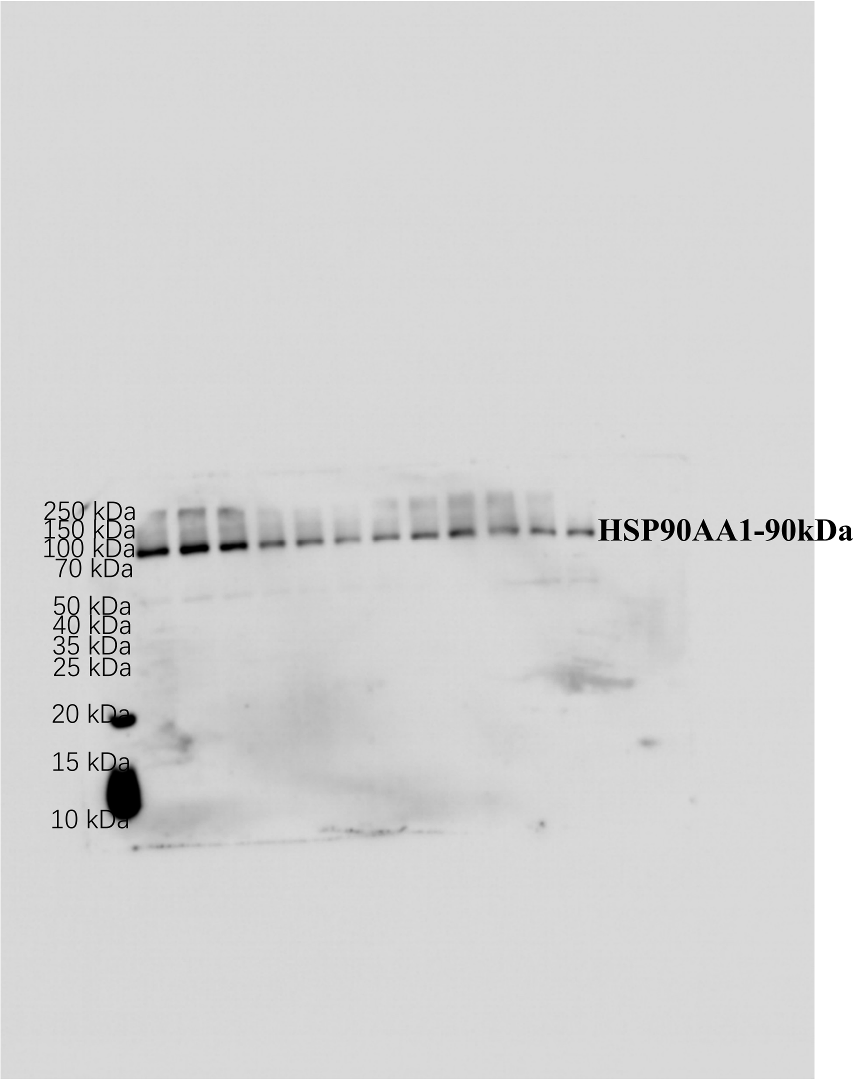

Figure 6D(CALR-55kDa)

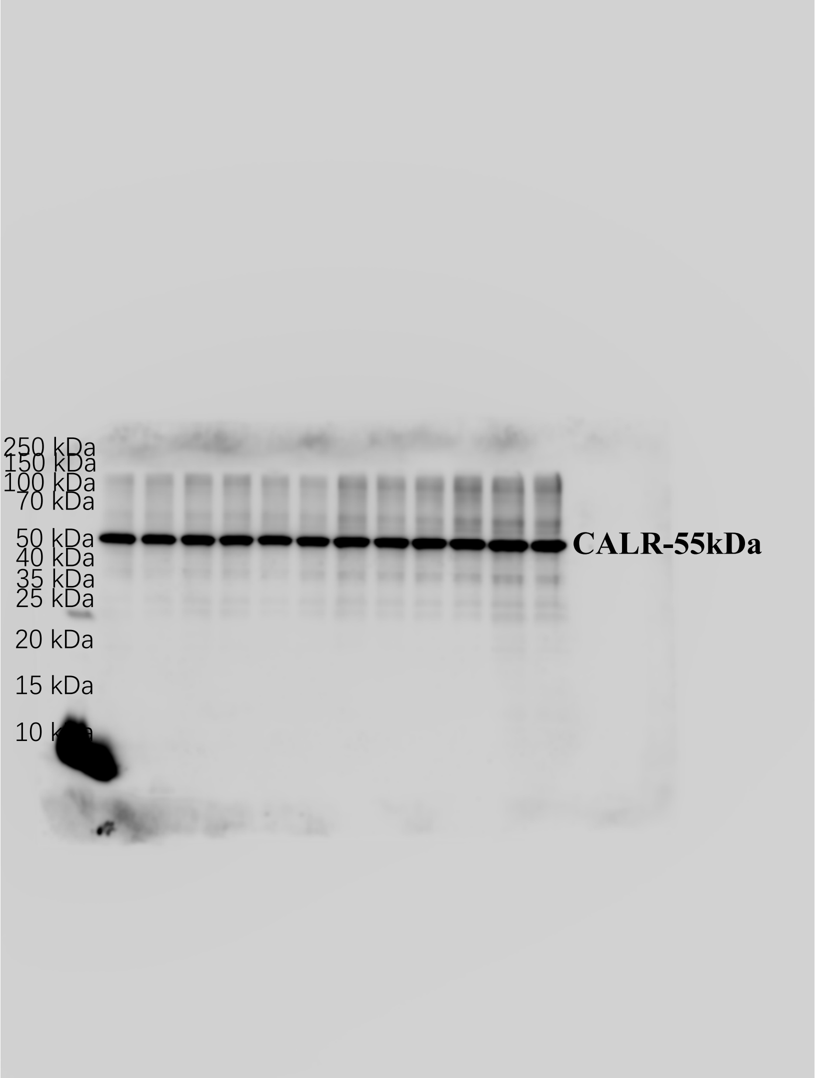

Figure 6D(HMGB1-25kDa)

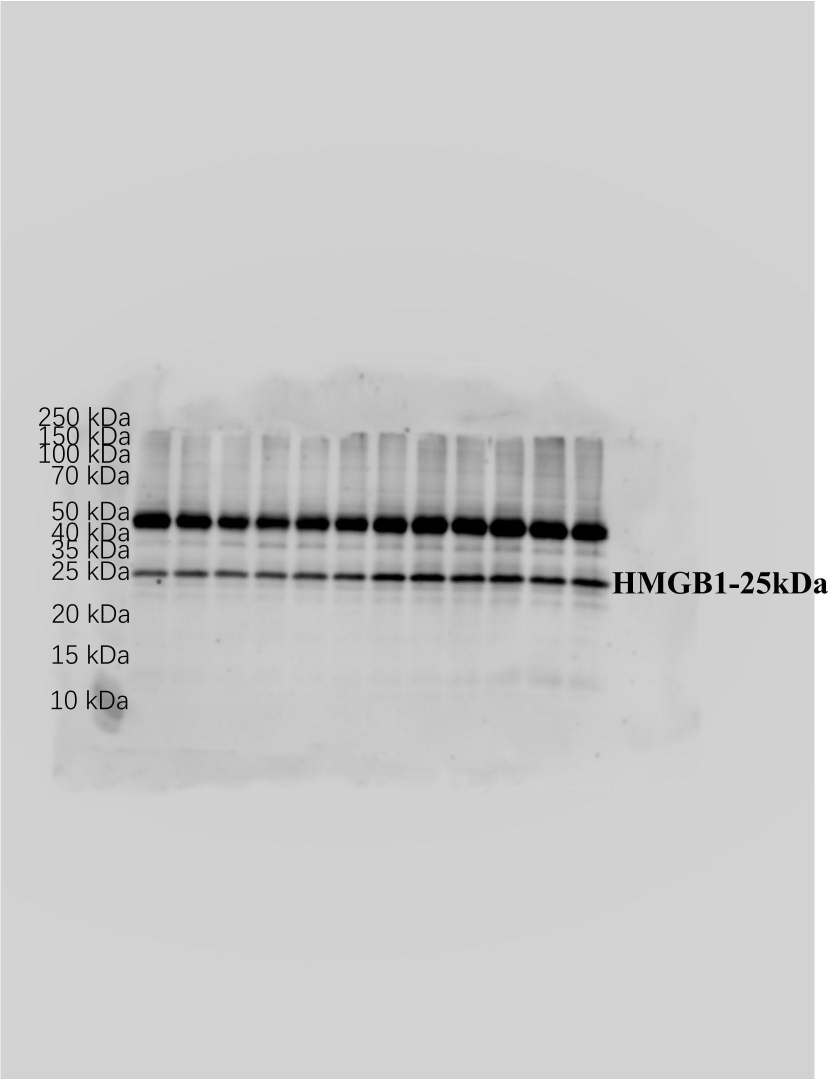

Figure 6D(CD8A-(25-32)kDa)

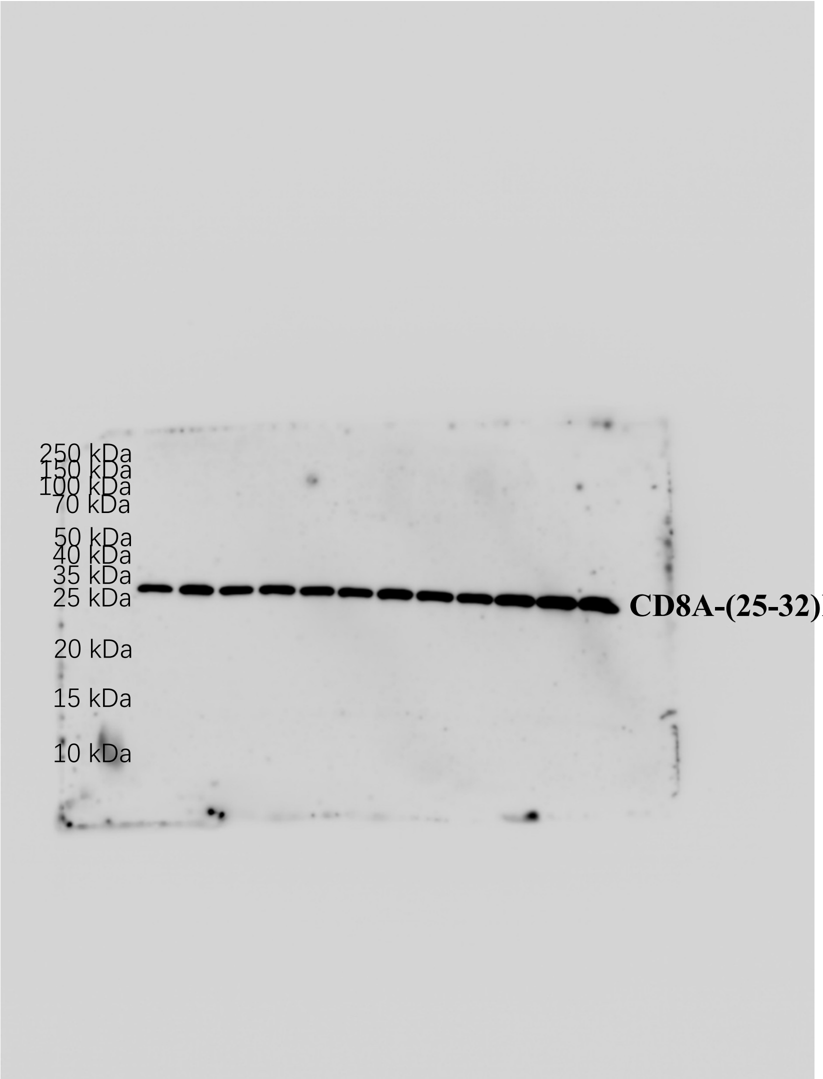

CD8A-(25-32)kDa

Figure 6D(GZMB-(28-32)kDa)

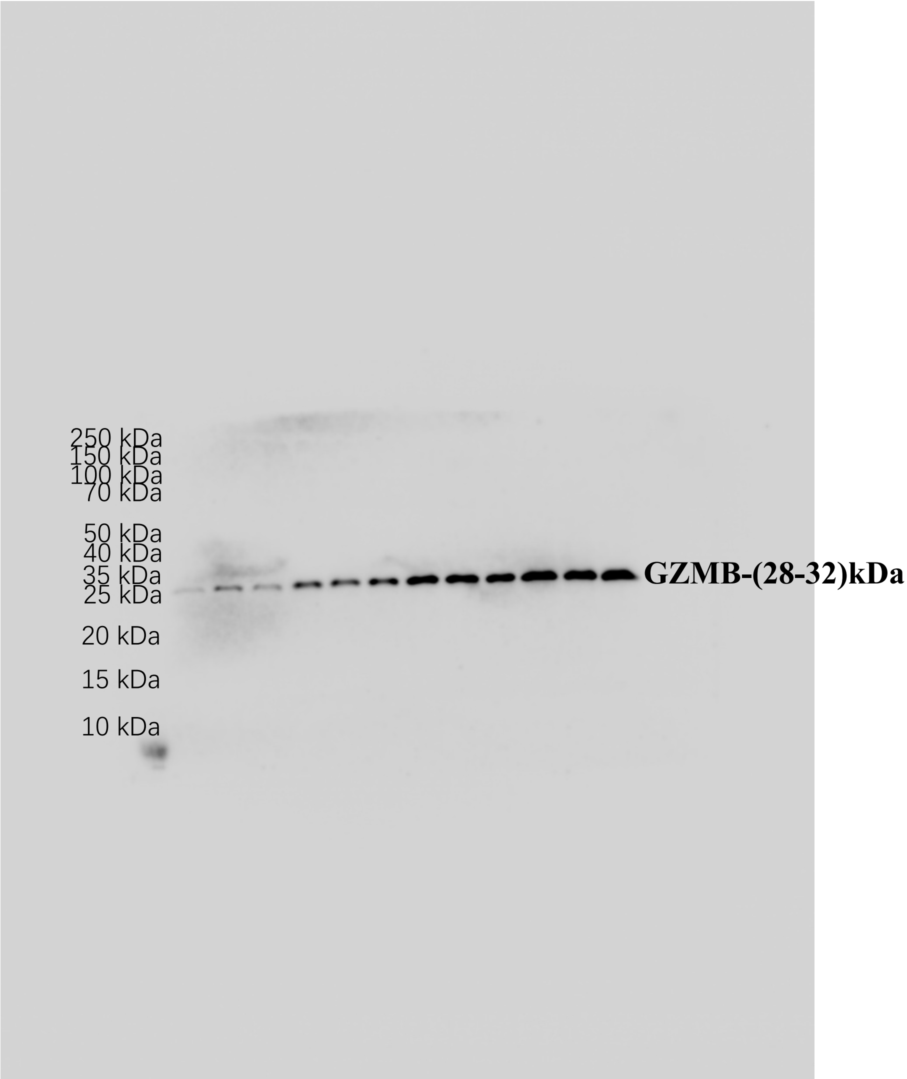

Figure 6D(PD-L1-(45-55)kDa)

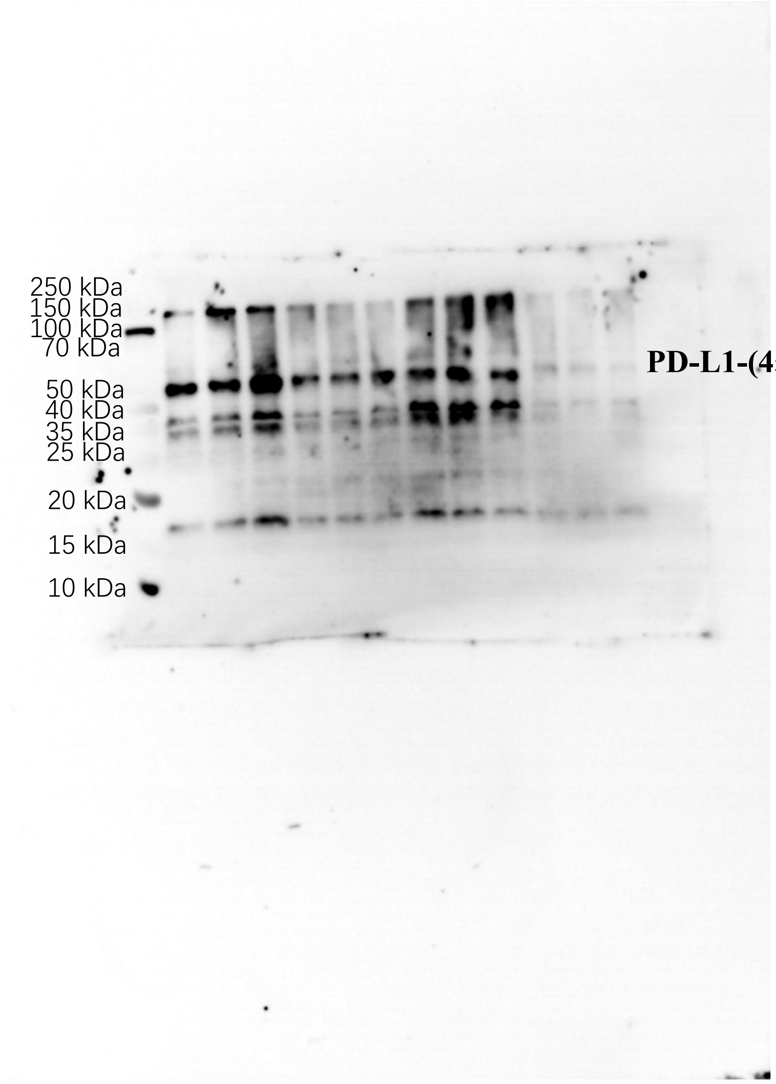

PD-L1-(45-55)kDa

Figure 6D(GAPDH-36kDa)

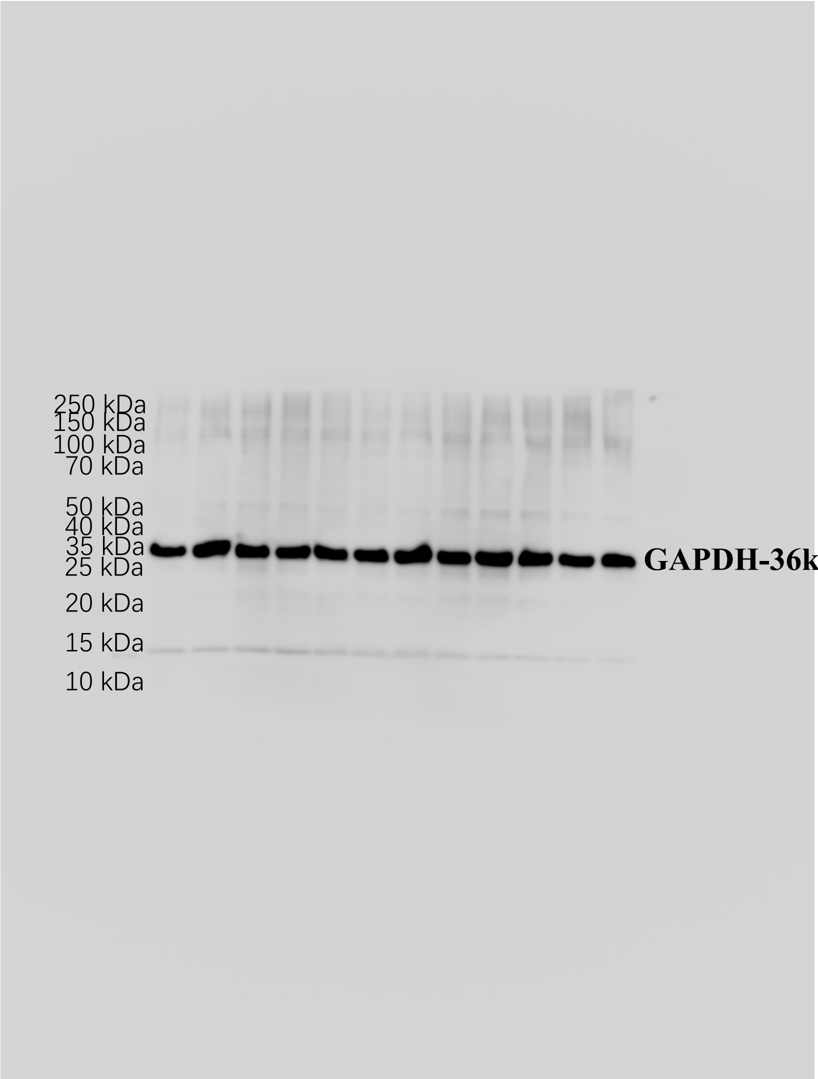

GAPDH-36kDa

Figure S3(HSP90AA1-90kDa)

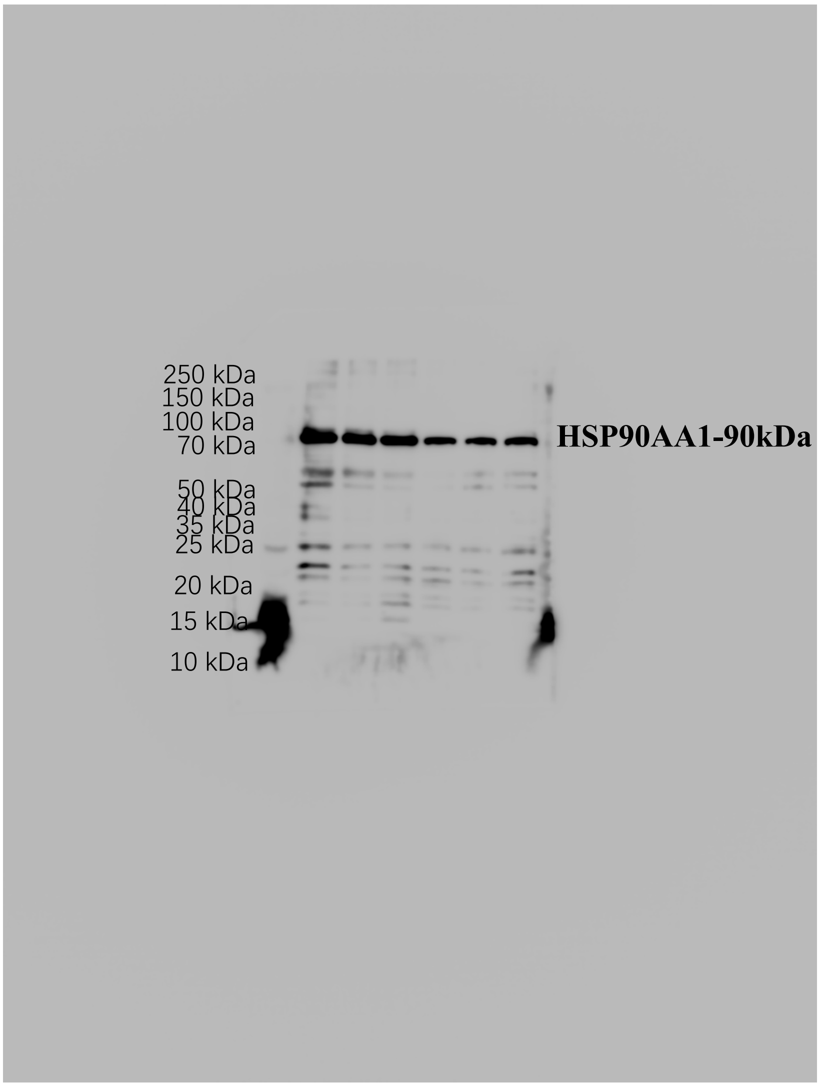

Figure S3(GAPDH-36kDa)

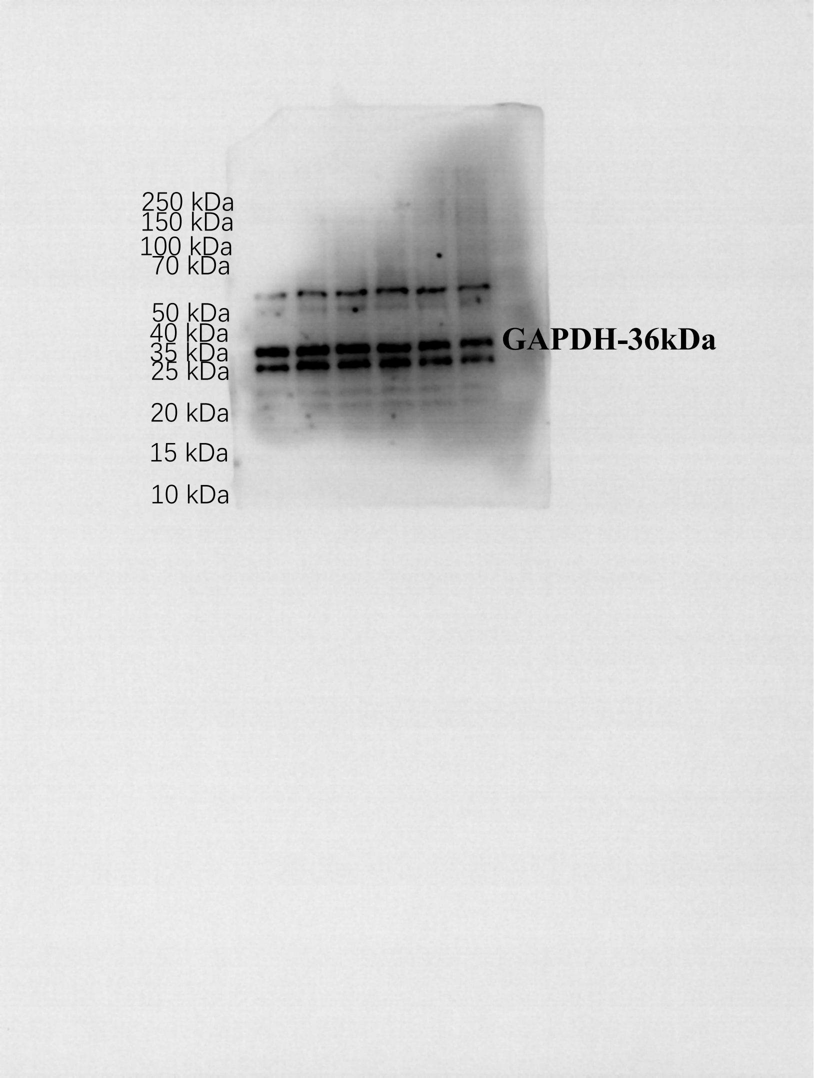

Supplement: Supplementary file 1 [file cancers-18-02913-s001.zip › File S1. WB raw data.pdf]
