# Supplementary material for: HSP90AA1 Knockdown Enhances Doxorubicin Sensitivity by Promoting Immunogenic Cell Death-Related Signaling and Immune-Related Tumor Microenvironment Remodeling in Breast Cancer
Source: Cancers (Basel). 2026 Sep 9;18(18):2913. doi: 10.3390/cancers18182913 (PMC13604569; doi:10.3390/cancers18182913)
Supplement: Supplementary file 1 [file cancers-18-02913-s001.zip › Table S1. Antibodies used for Western blotting.pdf]

Table S1. Antibodies used for Western blotting

Antibody	Host/Clonality	Working Dilution	Manufacturer/Location	Catalog No.
HSP90AA1	*rabbit polyclonal*	*1:1000*	*Proteintech, Rosemont, IL, USA*	*13171-1-AP*
Bax	*rabbit polyclonal*	*1:1000*	*Proteintech, Rosemont, IL, USA*	*23931-1-AP*
Bcl-2	*rabbit polyclonal*	*1:1000*	*Proteintech, Rosemont, IL, USA*	*12789-1-AP*
Cleaved-Caspase-3	*rabbit polyclonal*	*1:1000*	*Cell Signaling Technology, Danvers, MA, USA*	*9661*
CALR	*rabbit polyclonal*	*1:1000*	*Proteintech, Rosemont, IL, USA*	*10292-1-AP*
HMGB1	*rabbit polyclonal*	*1:1000*	*Proteintech, Rosemont, IL, USA*	*10829-1-AP*
eIF2α	*rabbit polyclonal*	*1:1000*	*Proteintech, Rosemont, IL, USA*	*11170-1-AP*
p-eIF2α	*rabbit polyclonal*	*1:1000*	*Proteintech, Rosemont, IL, USA*	*28740-1-AP*
CD8A	*rabbit polyclonal*	*1:1000*	*Proteintech, Rosemont, IL, USA*	*29896-1-AP*
GZMB	*rabbit polyclonal*	*1:1000*	*Proteintech, Rosemont, IL, USA*	*13588-1-AP*
FOXP3	*rabbit polyclonal*	*1:1000*	*Proteintech, Rosemont, IL, USA*	*22173-1-AP*
PD-L1	*rabbit polyclonal*	*1:1000*	*Proteintech, Rosemont, IL, USA*	*17952-1-AP*
GAPDH	*mouse monoclonal*	*1:10000*	*Proteintech, Rosemont, IL, USA*	*60004-1-Ig*
HRP-conjugated goat anti-rabbit IgG (H+L)	*goat polyclonal*	*1:10000*	*Proteintech, Rosemont, IL, USA*	*SA00001-2*
HRP-conjugated goat anti-mouse IgG (H+L)	*goat polyclonal*	*1:10000*	*Proteintech, Rosemont, IL, USA*	*SA00001-1*
